# Glycyrrhizic acid inhibits myeloid differentiation of hematopoietic stem cells by binding S100 calcium binding protein A8 to improve cognition in aged mice

**DOI:** 10.1186/s12979-023-00337-9

**Published:** 2023-03-11

**Authors:** Xue Gong, Haitao Shen, Liuling Guo, Ce Huang, Tingting Su, Hao Wang, Shengyu Feng, Shanshan Yang, Fenjiao Huo, Haifeng Liu, Jianbo Zhu, Jian-Kang Zhu, Hongbin Li, Hailiang Liu

**Affiliations:** 1 Key Laboratory of Xinjiang Phytomedicine Resource and Utilization of Ministry of Education, College of Life Sciences, Shihezi University, Shihezi, 832003 China; 2 Institute for Regenerative Medicine, Shanghai East Hospital, Tongji University School of Medicine, Shanghai, 200123 China; 3 China Colored-Cotton (Group) Company, Ltd, Urumqi, 830000 Xinjiang China; 4 Institute of Advanced Biotechnology, Southern University of Science and Technology, Shenzhen, 518055 China

**Keywords:** Glycyrrhizic acid (PubChem CID:14,982), Hematopoietic stem cells, Myeloid lineage, S100A8, Cognitive level

## Abstract

**Background:**

Glycyrrhizic acid (GA), a saponin compound often used as a flavoring agent, can elicit anti-inflammatory and anti-tumor effects, and alleviate aging. However, the specific mechanism by which GA alters immune cell populations to produce these beneficial effects is currently unclear.

**Results:**

In this study, we systematically analyzed single-cell sequencing data of peripheral blood mononuclear cells from young mice, aged mice, and GA-treated aged mice. Our in vivo results show that GA reduced senescence-induced increases in macrophages and neutrophils, and increased numbers of lymphoid lineage subpopulations specifically reduced by senescence. In vitro, GA significantly promoted differentiation of Lin^−^CD117^+^ hematopoietic stem cells toward lymphoid lineages, especially CD8^+^ T cells. Moreover, GA inhibited differentiation of CD4^+^ T cells and myeloid (CD11b^+^) cells by binding to S100 calcium-binding protein 8 (S100A8) protein. Overexpression of S100A8 in Lin^−^ CD117^+^ hematopoietic stem cells enhanced cognition in aged mice and the immune reconstitution of severely immunodeficient B-NDG (NOD.CB17-Prkdcscid/l2rgtm1/Bcgen) mice.

**Conclusions:**

Collectively, GA exerts anti-aging effects by binding to S100A8 to remodel the immune system of aged mice.

**Supplementary Information:**

The online version contains supplementary material available at 10.1186/s12979-023-00337-9.

## Background

Aging manifests itself in cells, tissues, organs, and the whole animal body, ultimately leading to a decline in body function and dysregulation of homeostasis. Senescence contributes to cell cycle arrest and produces a senescence-associated secretory phenotype (SASP) [1]. SASP factors recruit immune cells including macrophages, neutrophils, natural killer (NK) cells, and T cells to promote inflammatory responses [2]. SASP transmits senescence to adjacent cells and some upregulated inflammatory factors have a significant destructive effect on health organisms [3], or even trigger fatal degenerative diseases, such as Alzheimer's disease [4], diabetes [5], and cancer [6]. Mice exhibited signs of premature aging when only the immune system was allowed to age, and transplantation of young immune cells to prematurely aged mice produced a correspondingly delayed aging phenotype [7]. Release of the calreticulin-like protein grancalcin by senescent immune cells promotes skeletal aging, which reduces the quality of life of elderly individuals [8]. Moreover, the accumulation of age-related senescent cells shortens the lifespan and health of mice [9]. Both healthy long-lived seniors and long-lived mice exhibit good immune function, with immune cells displaying adequate oxidative and stress responses, and low expression of pro-inflammatory genes [10, 11]. Substantial evidence indicates that the immune system has important roles in aging and is inextricably linked to age-related diseases.

Glycyrrhizic acid (GA), an oleanolane-type pentacyclic triterpenoid saponin compound [12], is often used as a flavoring in chocolate, chewing gum, some alcoholic beverages, and cigarettes. It can constitute up to 15% of licorice root and is one of the most important active ingredients in licorice [13]. GA has anti-inflammatory, anti-tumor, antioxidant, antiviral, antibacterial, and immunomodulatory activities [14–17]. In addition, GA has good anti-inflammatory effects on inflammatory responses caused by age-related diseases. Postoperative cognitive impairment produces neuroinflammatory and Alzheimer's disease-related pathological conditions. Cognitive impairment of rats was improved by pretreatment with GA before surgery, as were neuroinflammation [expression of interleukin (IL)-1β, IL-6, tumor necrosis factor (TNF)-α, and nuclear factor κB (NF-κB)] and Alzheimer's disease-related pathology (Tau phosphorylation of AT-8, Ser369, and Aβ_40–42_) in the hippocampal region [18]. GA has shown good neuroprotective effects in neurodegenerative diseases and can improve cognition. So, is there some inextricable link between GA and enhancement of healthy aging? Our previous transcriptomic profiling of the blood of GA-treated aging female C57BL/6 mice showed that GA slowed aging associated with hematopoietic cell lineages. Moreover, GA could improve the cognitive ability of mice by increasing numbers of B and T cells, as verified in severely immunodeficient mice [19]. Therefore, this study further investigated the molecular mechanism by which GA improves cognition through the immune system to retard aging. Our findings provide a theoretical basis for applying small molecules and Traditional Chinese Medicine to the development of anti-aging drugs.

## Results

### Glycyrrhizic acid improved senescence-induced lymphocytopenia and myeloid cell increases

**Fig. 1:**
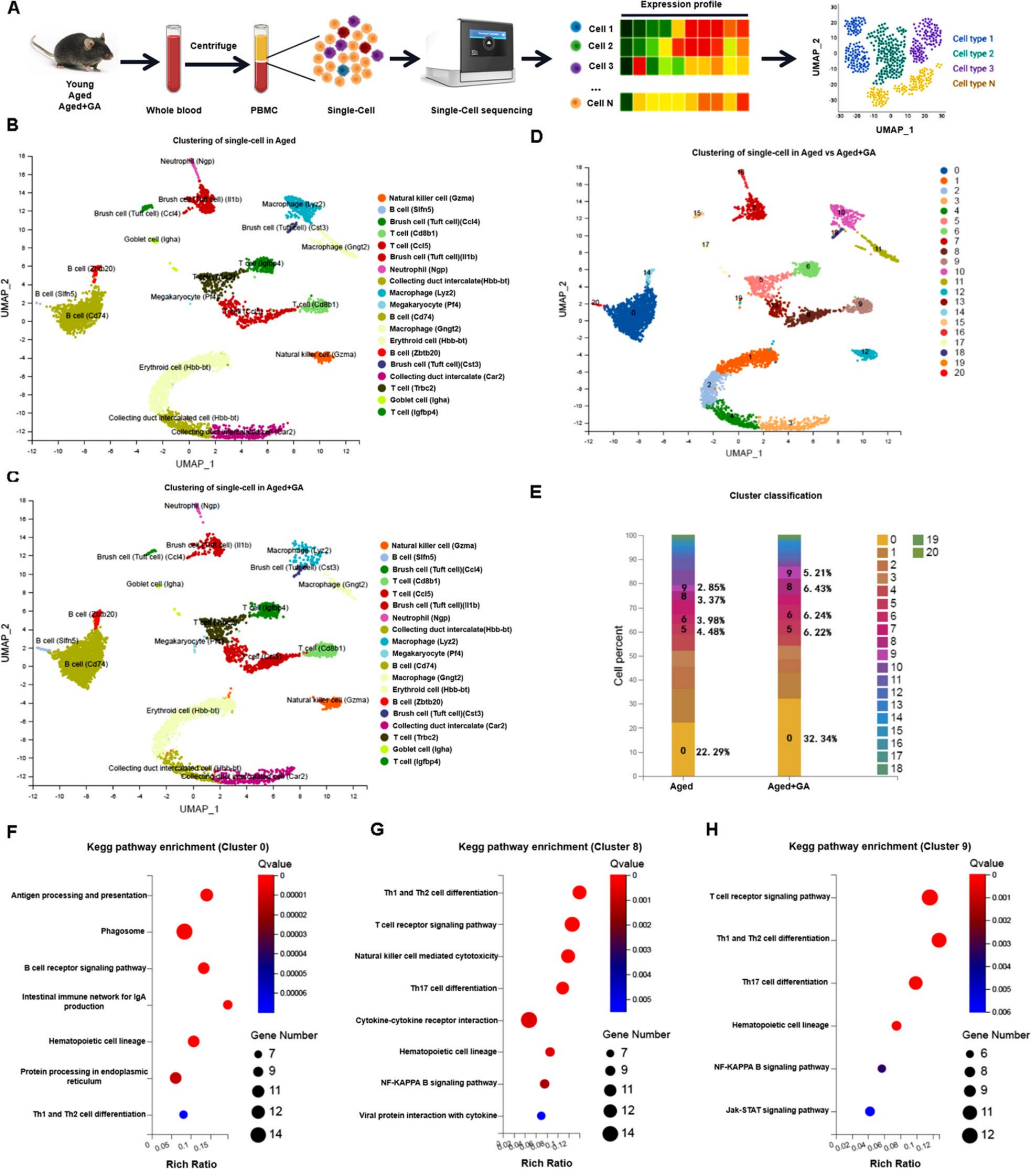
Single-cell clustering analysis of PBMCs from mice to evaluate changes in immune cell proportions during GA treatment. **A** Schematic of the experimental design for single-cell sequences. PBMCs collected from young (*n* = 5), aged (*n* = 5), and aged + GA (*n* = 5) mice were processed by scRNA-seq using a 10 × genomics platform. **B** Clustering of single cells in aged mice. **C** Clustering of single cells in aged + GA mice. **D** Comparison of clustering of single cells in aged and aged + GA mice. **E** Relative population abundances of cell types in aged and aged + GA mice. **F** KEGG analysis of differentially expressed genes in Cluster 0 (B cells, *Cd74* as marker gene). **G** KEGG analysis of differentially expressed genes in Cluster 8 (T cells, *Ccl5* as marker gene). **H** KEGG analysis of differentially expressed genes in Cluster 9 (T cells, *Cd8b1* as marker gene)

To investigate the effects of GA on the immune system-especially immunosenescence-during aging, we performed single-cell sequencing analysis of peripheral blood mononuclear cells (PBMCs) from 8-week-old (young), 16-month-old (aged), and GA-administered 16-month-old (aged-GA, 5 mg/kg every 2 d, tail vein injection, lasts 30 days) mice (Fig. 1A). Sequencing data were processed by CellRanger software. Cells from individual samples were collected, and a total of 30,124 single cells (Young, 7577; Aged, 10981; Aged-GA, 11566) were collected for subsequent analysis. Using uniform manifold approximation and projection (UMAP), different immune cell lineages were distinguished and identified according to expression of typical marker genes, namely: T cells, B cells, NK cells, macrophages, neutrophils, brush cells (columnar cells without wireless hairs), erythrocytes, and cup cells, whereby subpopulations of the same cell type were divided according to varying expression of different marker genes. PBMCs from young and aged mice were subjected to single-cell clustering analysis to evaluate changes in immune cell proportions during aging (Fig. S1A-D). Compared with young mice, aged mice had fewer T cells (*Igfbp4* and *Cd8b1* as marker genes), basal cells (*Dapl1*), B cells (*Iglc1*), NK cells (*Gzma*), and macrophages (*Ifit3*); and more erythrocytes (*Hba-a1* and *Hbb-bt*), neutrophils (*S100A8*), macrophages (*Lyz2*, *Cst3*, and *S100A8*), brush cells (*Ccl4* and *Cst3*), and cupped cells (*Igha*). Moreover, the results show increased proportions of senescent myeloid cells and decreased proportions of senescent lymphocytes in aged mice. In particular, T cells expressing *Igfbp4* and *Cd8b1* as marker genes (Clusters 1 and 2, respectively), and basal cells with *Dapl1* as a marker gene (Cluster 7) were mainly enriched for genes associated with the T-cell receptor signaling pathway; Th1, Th2, and Th17 cell differentiation; and gap junctions (Fig. S1E-G).

Single-cell clustering of PBMCs from aged and aged-GA mice to evaluate changes in immune cell proportions revealed that GA treatment increased T cells (*Cd8b1* and *Igfbp4* as marker genes), B cells (*Slfn*, *Zbtb20*, and *Cd74*), and NK cells (*Gzma*); and decreased macrophages (*Lyz2* and *Gngt2*), brush cells (*Ccl4*, *Cst3*, and *IL1b*), erythrocytes (*Hbb-bt*), cupped cells (*Igha*), and neutrophils (*Ngp*) (Fig. 1B-E). Major pathways enriched in B cells (*Cd74*, Cluster 0), T cells (*Ccl5*, Cluster 8; and *Cd8b1*, Cluster 9) included B cell and T cell receptor signaling pathways; and Th1, Th2, and Th17 cell differentiation (Fig. 1F-H). Additionally, following GA treatment, cells were analyzed using Monocle to construct proposed temporal developmental trajectories (darker colors represent earlier development with more posterior temporal differentiation). Macrophages, neutrophils, and brush cells were proposed to develop with anterior temporal differentiation, T cells with intermediate developmental temporal differentiation, and B cells with the latest developmental differentiation (Fig. S2A-C). Moreover, S100A8 was mainly expressed in Stage 1 in Clusters 9 (Brush cells), 12 (macrophages), and 15 (neutrophils) (Fig. S2D). These results, together with the previous comparison between young and aged mice, suggest that GA reduces the increase in macrophages and neutrophils caused by senescence, and increases numbers of T and B cells specifically reduced by senescence.

### Glycyrrhizic acid promoted differentiation of HSCs toward lymphocytes in vitro

**Fig. 2:**
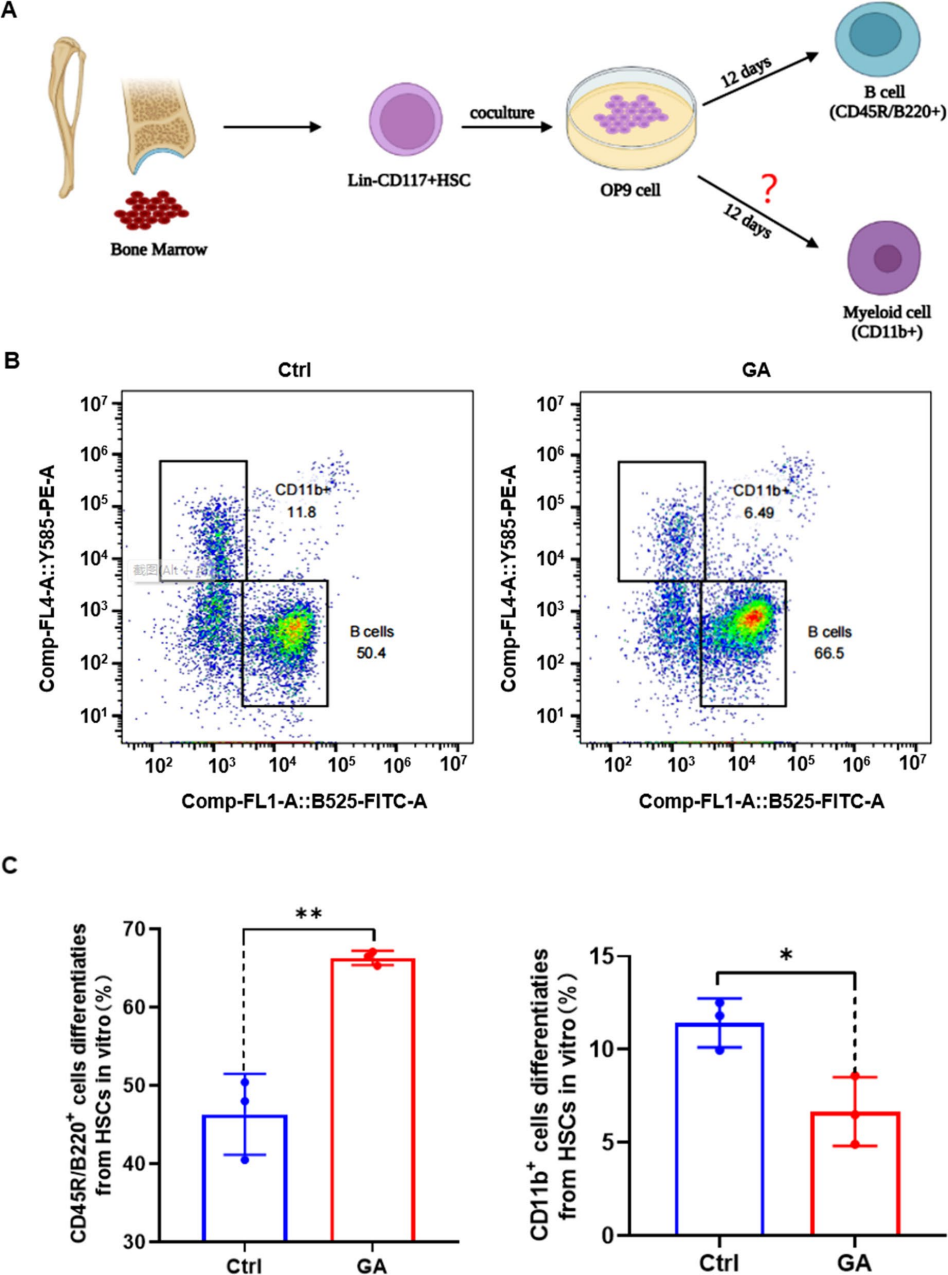
Glycyrrhizic acid promoted differentiation of Lin^−^CD117^+^ HSCs toward B cells in vitro. **A** Schematic design of Lin^−^CD117^+^ HSCs co-cultured with OP9 cells for directed differentiation to B cells. **B** Representative FACS plots of CD45R/B220^+^ B cells and CD11b^+^ cells following differentiation of Lin^−^CD117^+^ HSCs co-cultured with OP9 cells. **C** Bar graph of statistical results of CD45R/B220^+^ B cells and CD11b^+^ cells in co-cultures following differentiation of Lin^−^CD117.^+^ HSCs with OP9 cells. Data represent mean ± SEM. *n* = 3, **P < 0.01, *P < 0. 05

**Fig. 3:**
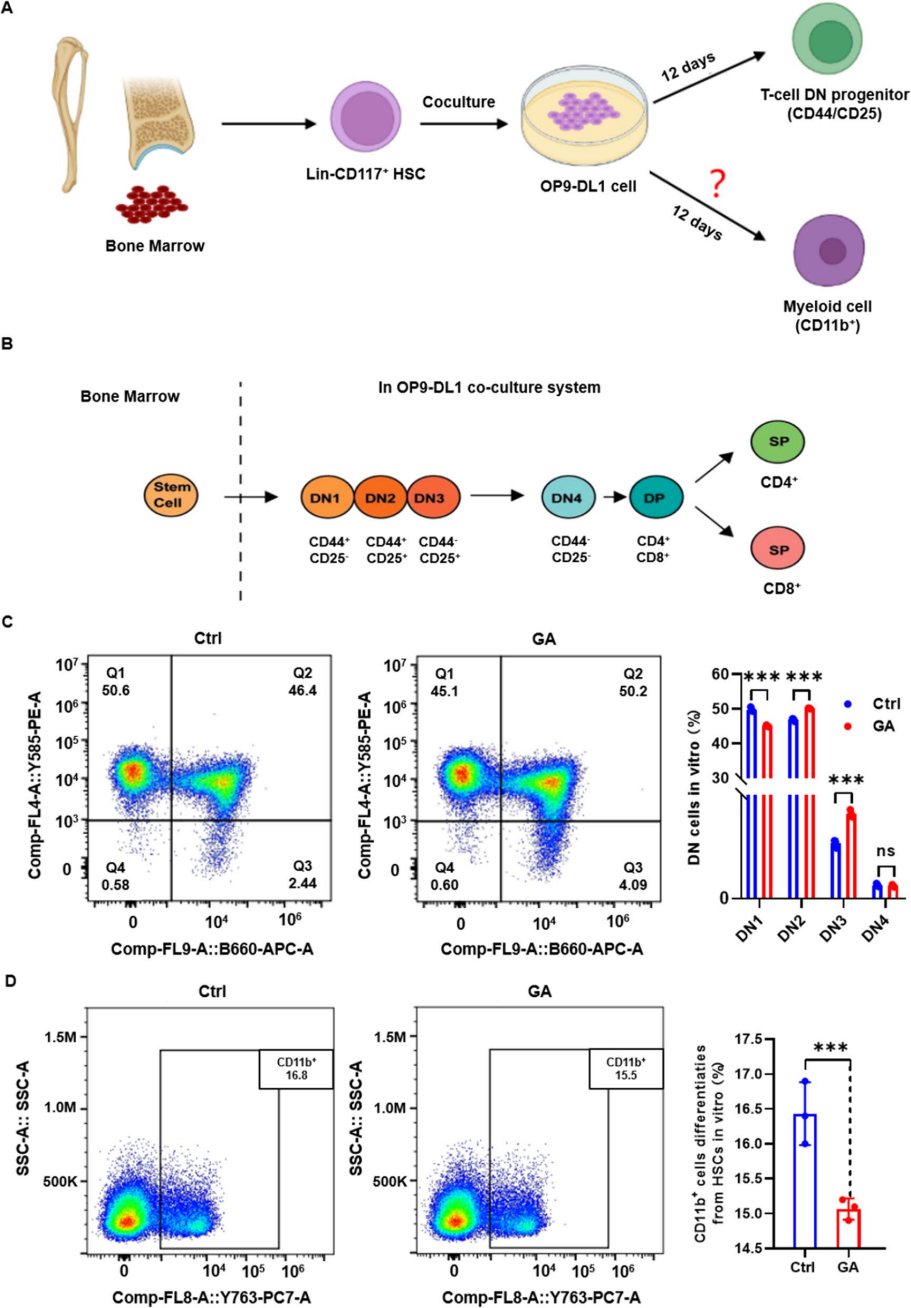
Glycyrrhizic acid promoted differentiation of Lin^−^CD117^+^ HSCs toward T cells in vitro. **A** Experimental design of Lin^−^CD117^+^ HSCs co-cultured with OP9-DL1 cells for short-term directed differentiation into T cells. **B** Schematic of the developmental maturation process of T cells by CD44 and CD25 negative–positive selection. **C** Representative FACS plots and bar graphs showing T-cell DN phase changes (DN1 phase, CD44^+^CD25^−^; DN2 phase, CD44^+^CD25^+^; DN3 phase, CD44^−^CD25^+^; DN4 phase, CD44^−^CD25^−^) following differentiation in co-cultures of Lin^−^CD117^+^ HSCs and OP9-DL1 cells. **D** Representative FACS plots and bar graphs of CD11b^+^ cells following co-culture differentiation of Lin^−^CD117^+^ HSCs and OP9-DL1 cells. Data represent mean ± SEM. *n* = 3, ***P < 0.001; ns, not significant vs. Ctrl

Single-cell data revealed that GA increased numbers of lymphatic lineage cells and decreased numbers of myeloid cells. On the basis of blood RNA sequencing in mice, the effect of GA was mainly associated with differential expression of genes in hematopoietic cell lineages, especially B and T cells [19]. First, the ability of GA to increase numbers of B and T cells was verified in vitro using spleen cells (Fig. S3), which proved consistent with our previous results [19]. Next, we further confirmed that GA increased numbers of B and T cells by promoting differentiation of hematopoietic stem cells (HSCs) toward the lymphoid lineage. Lin^−^CD117^+^ HSCs were purified and directed to differentiate toward lymphoid lineages (B and T cells) in vitro using an OP9/OP9-DL1 cell co-culture system. A directed B-cell differentiation model was established by co-culturing Lin^−^CD117^+^ HSCs with OP9 cells for 12 d (Fig. 2A). Subsequently, cells were analyzed by flow cytometry for markers of B cell (CD45R/B220^+^) and myeloid lineage cell (CD11b^+^) differentiation (Fig. 2B, C). The results show that GA promoted Lin^−^CD117^+^ HSC differentiation towards B cells; notably, although myeloid (CD11b^+^) cells were also produced in this co-culture system without the addition of myeloid differentiation factors, GA was able to inhibit myeloid cell production. A directed T cell differentiation model by co-culturing Lin^−^CD117^+^ HSCs with OP9-DL1 cells (Fig. 3A). T cells continued to differentiate into mature CD8^+^/CD4^+^ T cells after negative and positive selection for CD44 and CD25, respectively, and it was shown that a short period of directed T cell differentiation can only be observed in the DN phase [20] (Fig. 3B). Lin^−^CD117^+^ HSCs were co-cultured with OP9-DL1 cells for 12 d and then analyzed by flow cytometry for DN phase changes in T cells and myeloid (CD11b^+^) cell differentiation (Fig. 3C, D). The results show a significant decrease in the proportion of DN1-phase cells and significant increase in the proportion of DN2- and DN3-phase cells in the GA group, and no difference in DN4-phase cells between GA and control groups. These results suggest that GA drives DN phase changes of Lin^−^CD117^+^ HSCs during T-cell differentiation. Moreover, although myeloid (CD11b^+^) cells are also produced in this co-culture system without the addition of myeloid differentiation factors, the percentage of myeloid cells in the GA group was significantly reduced, indicating that GA suppressed myeloid cell production. The OP9/OP9-DL1 cell co-culture system inhibited production of myeloid (CD11b^+^) cells without the addition of myeloid differentiation factors.

**Fig. 4:**
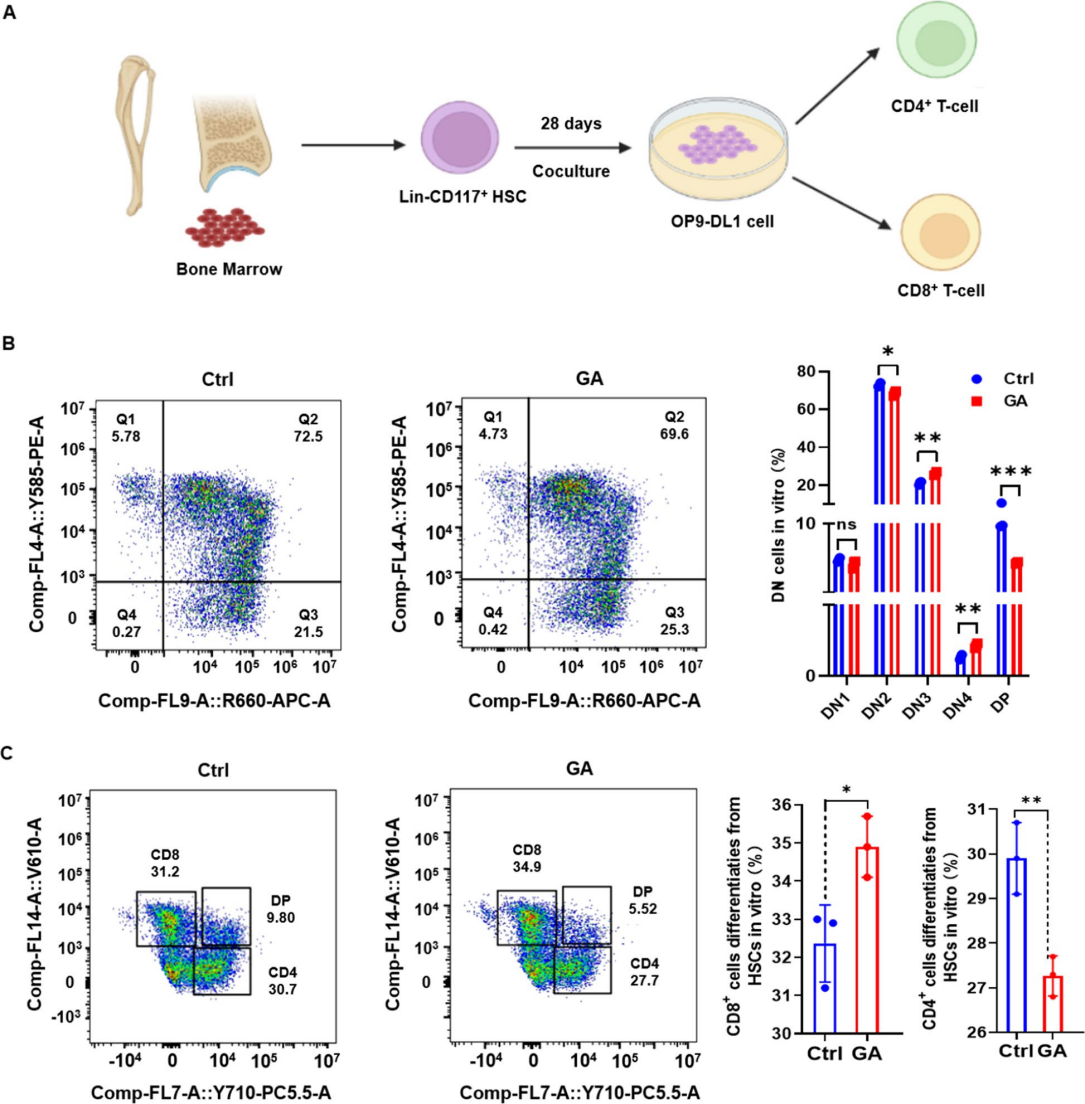
Glycyrrhizic acid promoted differentiation of Lin^−^CD117^+^ HSCs into CD8^+^ T cells in vitro. **A** Experimental design of Lin^−^CD117^+^ HSCs co-cultured with OP9-DL1 cells for long-term directed differentiation into T cells. **B** Representative FACS plots and bar graphs of T-cell DN phase changes (DN1 phase, CD44^+^CD25^−^; DN2 phase, CD44^+^CD25^+^; DN3 phase, CD44^−^CD25^+^; DN4 phase, CD44^−^CD25^−^; and DP phase, CD8^+^CD4^+^) in co-culture differentiation of Lin^−^CD117^+^ HSCs and OP9-DL1 cells. **C** Representative FACS plots and bar graphs of CD8^+^ T cells and CD4^+^ T cells in co-culture differentiation of Lin^−^CD117^+^ HSCs with OP9-DL1 cells. Data represent mean ± SEM. *n* = 3, *P < 0. 05, **P < 0.01, ***P < 0.001; ns, not significant vs. Ctrl

To observe the differentiation of mature CD8^+^/CD4^+^ T cells, a long-term (28 d) Lin^−^CD117^+^ HSC directed T cell differentiation model was established in vitro (Fig. 4A). Lin^−^CD117^+^ HSCs were co-cultured with OP9-DL1 cells for 28 d and then analyzed by flow cytometry for T-cell DN phase and expression of mature T-cell markers (CD8^+^/CD4^+^) (Fig. 4B, C). The results show that there was no significant difference in DN1-phase cells between GA and control groups, the proportion of DN2-phase cells was significantly decreased in the GA group, and proportions of cells in DN3 and DN4 phases were significantly increased in the GA group. Moreover, among the mature CD4^+^/CD8^+^ T cells generated, GA was able to significantly increase the number of CD8^+^ T cells and significantly decrease the number of CD4^+^ T cells. These results suggest that GA promoted the differentiation of Lin^−^CD117^+^ HSCs toward B cells and CD8^+^ T cells, and inhibited their differentiation toward CD11b^+^ myeloid cells, consistent with the results of PBMC single-cell sequencing. Collectively, these findings demonstrate that GA can improve the differentiation bias of HSCs caused by aging.

### S100A8 protein is the target protein of GA

**Fig. 5:**
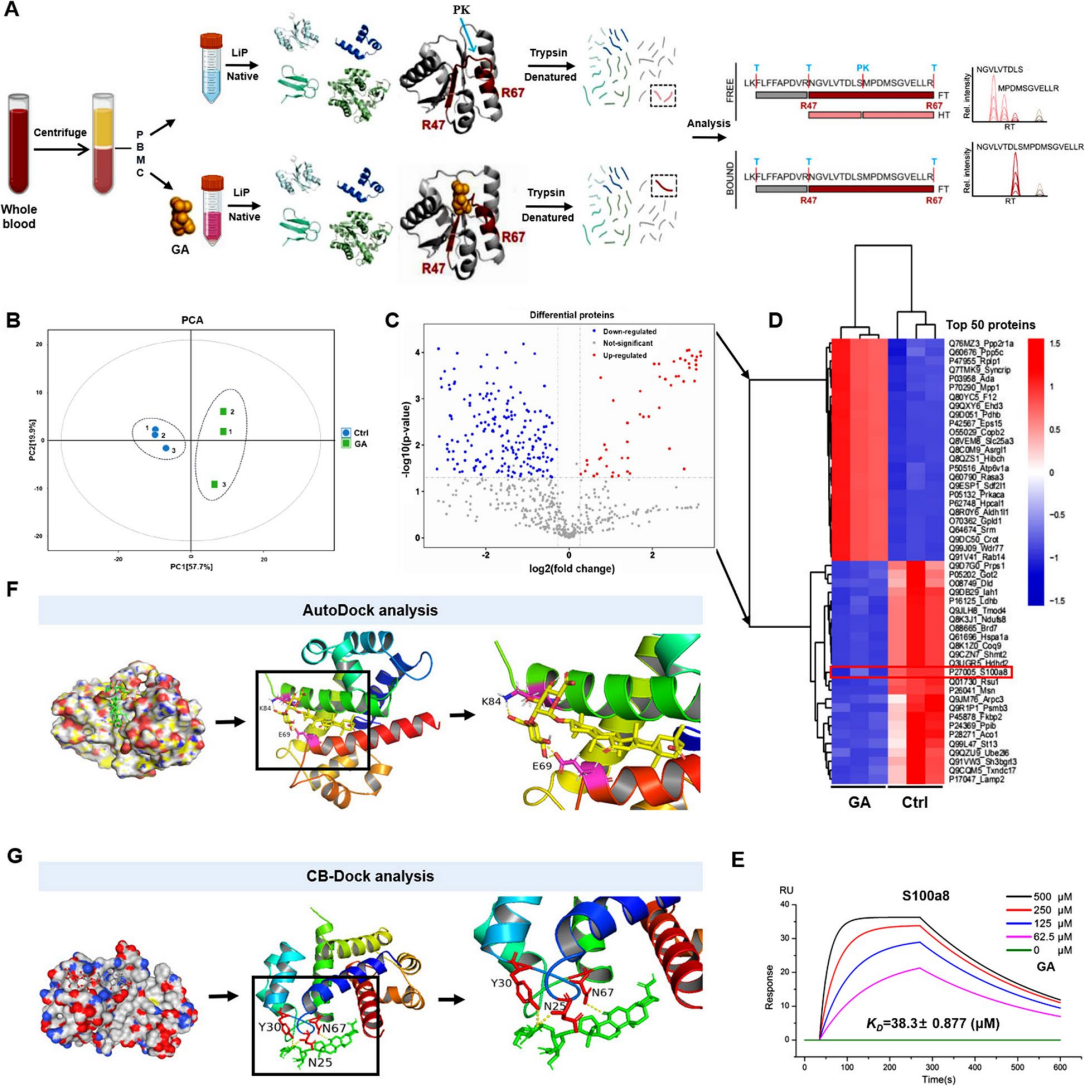
S100A8 protein binds GA. **A** Label-free quantitative (LFQ) proteomics study. Schematic diagram of experimental design for nanoLC-MS/MS analysis of GA and PBMC protein interaction. **B** QC plots for mass spectrometry samples, with no dispersion for each of the control and GA samples. **C** Differentially expressed peptides were screened according to the criteria: unique peptide ≥ 1, Foldchange > 1.2 or < 0.83, and *P*-value < 0.05. Three hundred differentially expressed proteins met these criteria. **D** Heatmap showing 50 most highly significant differentially expressed peptides in mass spectral results of GA versus control; S100A8 is the proposed binding protein for GA. **E** Binding affinity of SPR COOH and S100A8-GA interactions. S100A8 was immobilized on COOH chips with GA concentrations (from top to bottom) of 500, 250, 125, 62.5, and 0 μM. **F** AutoDock simulation of GA-S100A8 binding visualization map with hydrogen bond binding sites K84 and E69. **G** CB-Dock simulation of GA-S100A8 binding visualization map with hydrogen bond binding sites Y30, N67, and N25

To determine which proteins are targeted by GA to act in immune cells, we performed a label-free quantitative (LFQ) proteomics study. We extracted and purified PBMC proteins and GA-interacting PBMC proteins, then analyzed them using nanoLC-MS/MS (mass spectrometry) (Fig. 5A). After subjecting mass spectrometry samples to quality control (Fig. 5B), we detected a total of 829 proteins, which were digested by trypsin to obtain 5319 peptides with an enzymatic efficiency of 97.37%. Among them, differentially expressed proteins were screened according to the following conditions: unique peptide ≥ 1, Foldchange  >  1.2 or <  0.83, and *P*-value  <  0.05. There were 300 differentially expressed proteins that met these criteria (Fig. 5C). The 50 most significant differentially expressed proteins were selected for heat map analysis. Some of these proteins are involved in activation of the natural immune system, including S100A8 (Fig. 5D). Because immune cells all develop from HSCs, RNA-seq analysis showed that *S100A8* was one of several genes specifically upregulated in GA-treated Lin^−^CD117^+^ HSCs (Fig. S4A), which were mainly enriched in the IL-17 signaling pathway (Fig. S4B). Quantitative reverse transcription PCR (qRT-PCR) validation of differentially expressed genes *S100A8* and *S100A9* showed expression patterns consistent with RNA sequencing results of Lin^−^CD117^+^ HSCs (Fig. S4C, D). The results of single-cell sequencing of PBMCs shows that the mRNA expression level of S100A8 continuously increased with age and was reduced by GA treatment (Fig. S5). RNA sequencing results of the blood of mice treated with GA via tail vein injection showed an expression trend of *S100A8* [19] consistent with that of single cells. All of these results suggest that S100A8 is a likely target protein of GA. To further verify that GA bound to S100A8 protein, we analyzed this binding by surface plasmon resonance (SPR) molecular interactions. The experimental results show binding between GA and S100A8 protein (Fig. 5E).

We also simulated GA binding to S100A8 protein using CB-Dock and AutoDock simulation software. The protein structure of S100A8 was first predicted by homology modeling using SWISS-MODEL. The simulated protein structure of S100A8 protein and molecular structure of GA were loaded into CB-Dock and AutoDock for molecular docking. CB-Dock ran the docking program AutoDock Vina, and simulated binding results with high scores were selected according to Vina scores. In addition, AutoDock was able to identify binding sites with the highest affinity (according to binding energy) for the substrate and target protein with minimal global energy. The binding results with the highest affinity were visualized with PyMOL (Fig. 5G). Combining the results of these two-prediction software, we selected two possible binding sites near each other, N67 and E69, for further study. RNA-seq expression profiling analysis of MEFs overexpressing S100A8 (OEa8) or S100A8 with N67 and E69 point mutations (OEa8-67 and OEa8-69, respectively) revealed that OEa8-67 and OEa8-69 had 332 shared differential genes compared with OEa8. Gene Set Enrichment Analysis (GSEA) indicated significant enrichment of IL-17 signaling pathway genes among these differentially expressed genes (Fig. S6A) and indicated that these two amino acid sites were important for the function of S100A8 protein.

We also mutated S100A8 in MEFs with lentivirus-borne CRISPR/Cas9 technology and verified its mRNA expression level showed downregulation (Fig. S6B). RNA-seq expression profiling performed 48 h after adding GA showed that differentially expressed genes were mainly downregulated compared with control cells (Fig. S6C). Both KEGG pathway analysis showed enrichment of TNF-⍺ and IL-17 signaling pathways (Fig. S6D), while GSEA also indicated significant enrichment of the IL-17 signaling pathway (Fig. S6E). These results indicate that GA binds to S100A8 protein and the amino acid sites N67 and E69 are very important for S100A8 protein function.

### Overexpression of S100A8 inhibited differentiation of HSCs toward myeloid cells in vitro

**Fig. 6:**
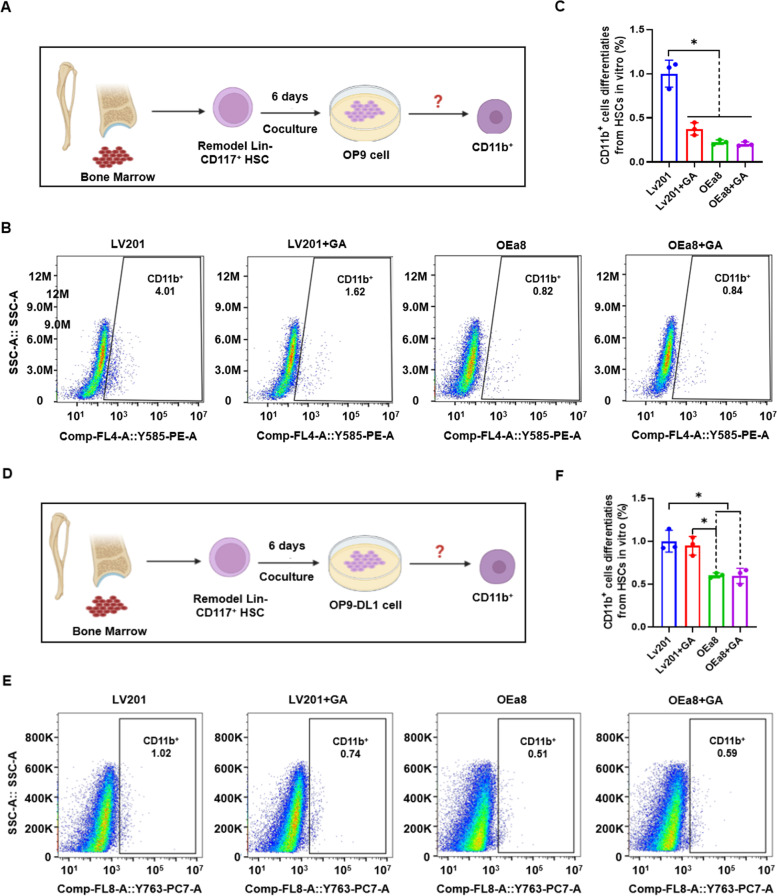
Co-culture differentiation of modified Lin^−^CD117^+^ HSCs with OP9/OP9-DL1 cells. **A** Experimental design of co-culture differentiation of Lin^−^CD117^+^ HSCs with OP9 cells for 6 d in LV201, LV201 + GA, OEa8 (LV201 + S100A8), and OEa8 + GA (LV201 + S100A8 + GA) groups. **B** Representative FACS plots of CD11b^+^ cells in co-cultures of Lin^−^CD117^+^ HSCs with OP9 cells in LV201, LV201 + GA, OEa8, and OEa8 + GA groups. **C** Bar graph showing CD11b^+^ cells in co-cultures of Lin^−^CD117^+^ HSCs with OP9 cells in LV201, LV201 + GA, OEa8, and OEa8 + GA groups. **D** Experimental design of co-culture differentiation of Lin^−^CD117^+^ HSCs with OP9-DL1 cells for 6 d in LV201, LV201 + GA, OEa8, and OEa8 + GA groups. **E** Representative FACS plots of CD11b^+^ cells in co-cultures of Lin^−^CD117^+^ HSCs with OP9-DL1 cells in LV201, LV201 + GA, OEa8, and OEa8 + GA groups. **F** Bar graph showing CD11b^+^ cells in co-cultures of Lin^−^CD117^+^ HSCs with OP9-DL1 cells in LV201, LV201 + GA, OEa8, and OEa8 + GA groups. Data represent mean ± SEM, *n* = 3, *P < 0.05

To explore the role of S100A8 in HSC differentiation, we induced differentiation of Lin^−^CD117^+^ HSCs toward lymphoid lineage cells in the presence of *S100A8* overexpression. Virus-infected Lin^−^CD117^+^ HSCs were co-cultured with OP9 cells for directed differentiation toward B cells (Fig. 6A) and myeloid lineage (CD11b^+^) cells. Differentiation was analyzed by flow cytometry after 6 d of co-culture (Fig. 6B, C). Myeloid lineage (CD11b^+^) cells were produced in this co-culture system without the addition of myeloid differentiation factors; however, there was a tendency for GA to suppress this differentiation, although this difference was not significant. Moreover, overexpression of *S100A8* inhibited myeloid (CD11b^+^) cell differentiation to some extent. Virus-infected Lin^−^CD117^+^ HSCs were co-cultured with OP9-DL1 cells for directed T-cell differentiation (Fig. 6D), and myeloid (CD11b^+^) differentiation was analyzed by flow cytometry (Fig. 6E, F). Myeloid (CD11b^+^) cells were also produced in this co-culture system without the addition of myeloid differentiation factors, and GA inhibited myeloid production, consistent with the results described above for directed T-cell differentiation. Overexpression of S100A8 further inhibited myeloid (CD11b^+^) cell production. These results demonstrate a correlation between *S100A8* and myeloid differentiation in that increasing *S100A8* expression inhibited the differentiation of Lin^−^CD117^+^ HSCs toward myeloid cells.

### Glycyrrhizic acid binding to S100A8 improved cognition through the immune system in mice

**Fig. 7:**
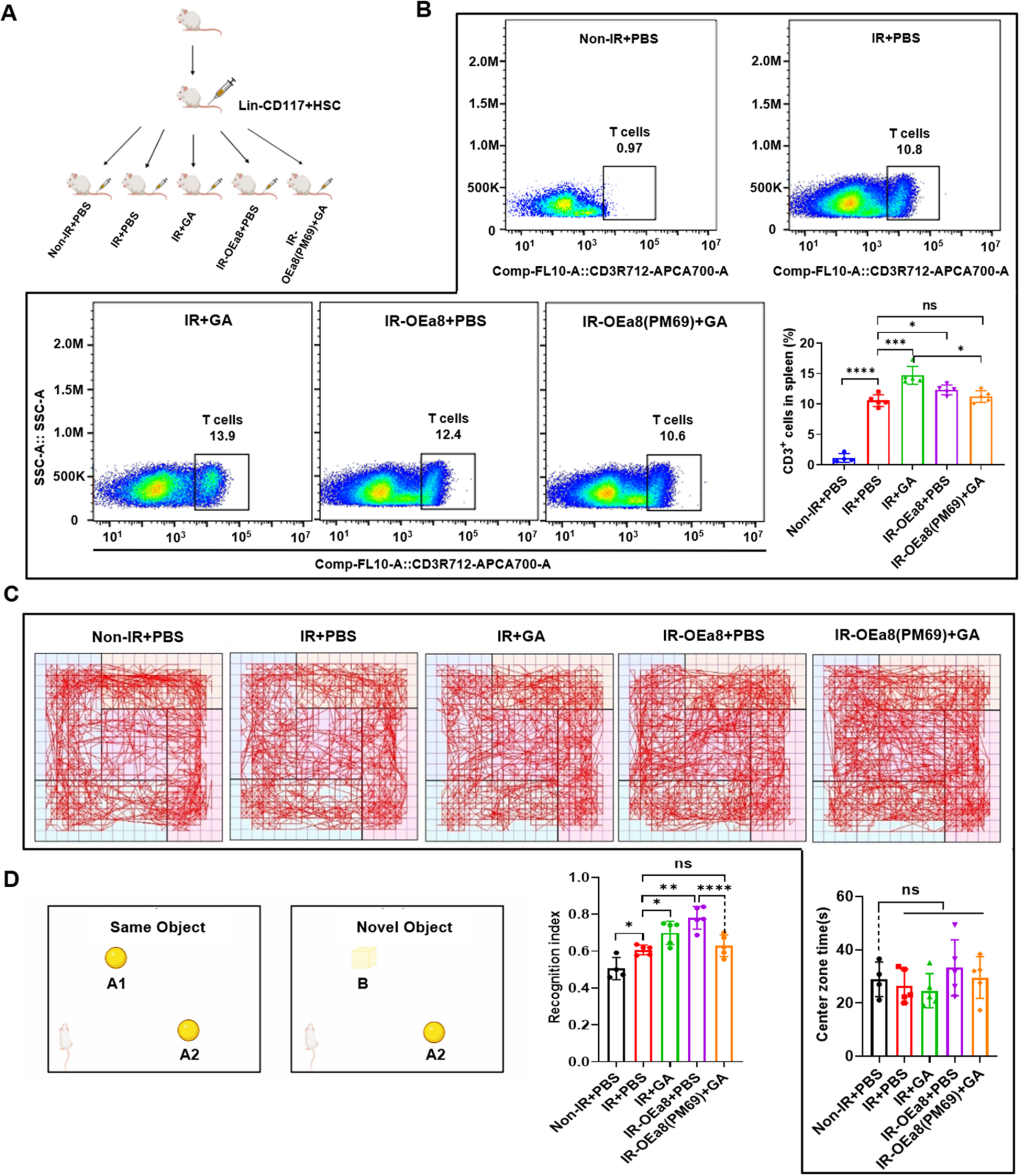
GA binding S100A8 improved cognitive levels of mice via the immune system. **A** Schematic design of immune reconstitution experiment in B-NDG mice with Lin^−^CD117^+^ HSCs, divided into the following groups: non-IR + PBS, IR + PBS (LV201 + PBS), IR + GA (LV201 + GA), IR-OEa8 + PBS (LV201-S100A8 + PBS), and IR-OEa8 (PM69) + GA (LV201-S100A8 (PM69) + GA. **B** Representative FACS plots and bar graphs showing CD3^+^ T cells in the spleens of mice in non-IR + PBS, IR + PBS, IR + GA, IR-OEa8 + PBS, and IR-OEa8 (PM69) + GA groups. **C** Walking paths tracked by software in non-IR + PBS, IR + PBS, IR + GA, IR-OEa8 + PBS, and IR-OEa8 (PM69) + GA groups of mice in the OFT. **D** Bar graphs showing times spent in the central area of the OFT in non-IR + PBS, IR + PBS, IR + GA, IR-OEa8 + PBS, and IR-OEa8 (PM69) + GA groups. **E** Experimental design of NOR. Bar graph of cognitive index statistics of mice in non-IR + PBS, IR + PBS, IR + GA, IR-OEa8 + PBS, and IR-OEa8 (PM69) + GA groups. Data represent mean ± SEM. non-IR + PBS, *n* = 4; IR + PBS, IR + GA, IR-OEa8 + PBS, and IR-OEa8 (PM69) + GA groups, *n* = 5, *P < 0. 05, **P < 0.01, ****P < 0.0001; ns, not significant vs. Ctrl

To observe the effect of GA binding to S100A8 through the immune system on the cognition of mice, we used lentivirally packaged Lin^−^CD117^+^ HSCs for immune reconstitution (IR) in severely immunodeficient B-NDG mice, and assessed the cognitive level of mice using open field test (OFT) and new object recognition (NOR) experiments. Lentiviral-packed Lin^−^CD117^+^ HSCs were administered to B-NDG mice by tail vein injection, and the IR and treatment groups were set up as follows: non-IR, IR + PBS, IR + GA, IR-OEa8 + PBS, and IR-OEa8 (PM69) + GA (Fig. 7A). We analyzed percentages of CD3^+^ T cells and CD45R/B220^+^ B cells by flow cytometry to observe the effect of IR. The results show that B-NDG mice were successfully reconstituted in that T cells and B cells were significantly increased in the IR + PBS group compared with those in the control group. The GA treatment group showed increased numbers of T cells and B cells compared with the reconstituted control group, and the IR-OEa8 + PBS group also displayed increased numbers of T cells and B cells; the IR-OEa8 (PM69) + GA group was similar to the reconstituted control group in T cells and B cells (Figs. 7B and S7). On the basis of the times mice spent in the central area during the 15-min OFT (Fig. 7C), there was no significant difference between experimental groups, indicating that the mice did not develop anxiety. NOR, which uses the instinctive exploration of novel objects to detect the fineness and sensitivity of a mouse’s recognition memory, was performed in mice without depression. Using a discrimination index to quantify the cognitive level of mice (Fig. 7D) revealed that reconstituting the immune system could improve the cognitive level of mice. Moreover, GA further improved the cognitive level of mice on the basis of immune reconstruction. The cognitive level of mice overexpressing S100A8 was substantially improved, even higher than that of the GA administration group. However, mice overexpressing *S100A8* with a point mutation (OEa8-PM69) that were treated with GA displayed significantly impaired cognitive levels. Collectively, these results suggest that GA can bind S100A8 to improve cognition through the immune system in all groups of mice without anxiety, and this effect is partially eliminated by mutating the GA binding site in S100A8.

## Discussion

GA has medicinal activities such as anti-inflammatory, anti-tumor, and immunomodulatory effects. In a study conducted by our group in the last 2 years, GA was found to improve aging and enhance cognition in aging mice by promoting the proliferation of B and T cells [19]. The role of GA in anti-aging was exhibited, but the mechanism underlying the anti-aging action of GA was still unclear. Sequencing the blood of aging mice revealed that the pathway through which GA acts is enriched in hematopoietic cell lineages, so are these increased numbers of B and T cells caused by HSC differentiation? Thus, we directed B- and T-cell directional differentiation in vitro using OP9 and OP9-DL1 co-culture system [20–22], respectively, and found that GA drives cell differentiation toward lymphoid lineages at the level of HSC differentiation. In the absence of added cytokines and culture conditions for differentiation into myeloid lineages, myeloid (CD11b^+^) cells were still produced, and GA inhibited the production of these cells. With age, the function of HSCs decreases and their potential for differentiation into blood cells of different lineages is biased, showing increased differentiation of myeloid cells and decreased differentiation of lymphocytes [23–25]. This was similarly verified in our 10× single-cell sequencing, wherein populations of T, B, and NK cells in the lymphocyte lineage were reduced in senescent mice compared with those in young mice, while populations of neutrophils and macrophages (myeloid cell lineages) were increased. The observed decrease in T, B, and NK cells and increase in neutrophils and macrophages were ameliorated after tail vein injection of GA, complementing our in vitro findings. "Immunosenescence" has become a hot angle in the study of aging. Robinson et al. selectively deleted *Ercc1*, which encodes a DNA repair protein, in mouse HSCs to construct a mouse model with only immune cell senescence, in which mice entered adulthood healthy and showed premature aging [26], further demonstrating that GA is able to retard aging through the immune system.

S100A8, also known as MRP-8, is a small 12-kDa protein from the S100 protein family. The known regulator of S100A8 is P53, which plays a decisive role as an inflammatory factor in the development of inflammation and tumors [27, 28]. Under physiological conditions, S100A8 exerts its biological function by forming S100A8/S100A8 homodimers in the cytoplasm through covalent bonding, or S100A8/S100A9 heterodimers with S100A9 in a Ca^2+^-dependent manner. Although Ca^2+^-dependent tetramer formation of S100A8 and S100A9 are essential for biological activity, homodimers rarely exist because of stability. Both S100A8 and S100A9 have helix-loop-helix motifs of charged amino acid residues, resulting in their high affinity for divalent ions. Thus, divalent ions alter the conformation of S100A8/S100A9, allowing them to perform their corresponding functions. S100A8/S100A9 can stimulate TNF-⍺ and IL-6 production in BV-2 microglia through ERK/NF-κB and JNK/NF-κB signaling pathways [29]. Among the multiple inflammatory pathways mediated by Toll-like receptor 4 and receptor for advanced glycation end-products (RAGE), S100A8/S100A9 plays an important role in protecting individuals from curative infections [30]. However, our findings reveal that GA can also bind to S100A8 protein. First, S100A8 was immobilized on a COOH chip, which captured S100A9 binding to S100A8 (Fig. S8A). However, GA affected the binding of S100A8 to S100A9 to some extent after first binding to S100A8 (Fig. S8B). Therefore, there is a competitive binding relationship between GA and S100A9 in binding of S100A8, and GA may play a biological function by blocking the binding of S100A8 to S100A9, although the specific mechanism needs further study.

In conclusion, mRNA expression of S100A8 in GA-treated normal HSCs is elevated and increases CD8^+^ T cells while decreasing CD4^+^ T cells. The absence of S100A8 in hematopoietic stem progenitor cells in a specific tumor microenvironment significantly diminished the number of CD8^+^ T cells [31] and affected the function of CD4^+^ T cells to some extent, which has some similarity with our results. Additionally, S100A8 can induce T-cell immune tolerance in specific tumor-bearing microenvironments, but no relevant studies have been able to demonstrate an effect of S100A8 on CD8^+^ T-cell chemotaxis and function. Deletion of S100A8 significantly increased numbers of myeloid-derived suppressor cells in bone marrow and spleen, even when not in specific tumor-bearing microenvironments but the natural state. In the first known *S100A8*^−/−^ mouse, there was a significant increase in numbers of myeloid cells, including neutrophils, monocytes, and dendritic cells, distributed in the peripheral blood and bone marrow [32]. In acute myeloid leukemia, S100A8 not only inhibits S100A9-induced terminal differentiation of acute myeloid leukemia (AML) cells, but also favors AML growth. Moreover, S100A8-knckout mice exhibit significant increases in myeloid cells (especially neutrophils, monocytes, and their precursors) in the bone marrow [33]. Furthermore, evidence suggests that downregulation of S100A8 restored erythroid lineage differentiation in a mouse model of myelodysplastic syndrome [34]. Overexpression of S100A8 in HSCs, whereby modified HSCs are subject to long-term differentiation and can therefore only differentiate for short periods of time, we observed changes in proportions of CD11b^+^ cells, and overexpression of S100A8 protein reduced CD11b^+^ cell production. This evidence suggests that S100A8 is inextricably linked to myeloid cell generation.

## Methods

### Animals

C57BL/6 mice (8W) were purchased from Shanghai SLAC Laboratory Animal (Shanghai, China). Aged C57BL/6 mice (16 months) were from laboratory-reared reserves. B-NDG severely immunodeficient mice (8 weeks) were purchased from Beijing Biocytogen (Beijing, China). All mice used in experiments were female and housed in the specific-pathogen-free-grade laboratory animal center of Tongji University School of Medicine. Mice were housed five per cage, maintained on a 12-h light/dark cycle, at an appropriate temperature, with free access to water and food. Animal care and procedures for this study were in accordance with institutional guidelines and the Animal Welfare Act, and all protocols involving experimental animals were approved by Experimental Animal Ethics Committee of Tongji University School of Medicine. Mice of equal weights in each age group were randomly grouped. GA was administered every other day by tail vein injection, and the same dose of phosphate-buffed saline (PBS) was injected into the tail vein of the control group.

### 10 × Single-cell sequencing

PBMCs were isolated from the blood of mice. Cell viability was examined under a microscope with 0.4% trypan blue staining. When the survival rate of cells reached 80% or more, library construction experiments were performed. Single-cell libraries were constructed using Chromium™ Controller and Chromium™ Single Cell 3ʹ Reagent Version 3 Kit (10 × Genomics, Pleasanton, CA). Single cells, reagents, and gel beads were enclosed in “gel beads in emulsion” (GEMs). Lysis and barcoded reverse transcription of single-cell polyadenylate mRNA was performed within each GEM. RT-GEMs were cleaned up to amplify cDNA, which was subsequently fragmented. Fragmented ends were repaired and A-tailing was added at the 3' end. Aptamers were ligated to fragments that were screened by two-sided solid-phase reversible immobilization (SPRI). After sample index PCR, another two-sided SPRI screen was performed. The final library was checked for fragment size distribution using an Agilent 2100 Bioanalyzer (Santa Clara, CA) and the library was quantified using real-time quantitative PCR with TaqMan probes. Finally, sequencing was performed using the DNBseq platform (BGI Group, Shenzhen, China).

### Analysis of single-cell transcriptomics data

Single-cell FASTQ sequencing reads from each sample were processed and converted to digital gene expression matrices after mapping to the reference genome using the Cell Ranger Single Cell Software Suite (v3.1.0) [35] provided on the 10 × genomics website. Individual datasets were aggregated using the CellRanger aggr command without subsampling normalization. The aggregated dataset was then analyzed using the R package Seurat (v 3.1.0) [36]. First, the dataset was trimmed of cells expressing fewer than 200 genes. Next, the number of genes, UMI counts, and percentage of mitochondrial genes were examined to identify outliers. Because an unusually high number of genes can result from a ‘doublet’ event, in which two different cell types are captured together within the same barcoded bead, cells with > 90% of maximum genes were discarded. Cells containing > 7.5% mitochondrial genes were presumed to be of poor quality and also discarded. Next, gene expression values underwent library-size normalization, in which raw gene counts from each cell were normalized relative to the total number of read counts present in that cell. The resulting expression values were then multiplied and log-transformed. Subsequent analyses were conducted using only the most highly variable genes in the dataset. Principal component analysis was used for dimensionality reduction, followed by clustering in principal component analysis space using a graph-based clustering approach. Uniform Manifold Approximation and Projection (UMAP) was then used for two-dimensional visualization of the resulting clusters. For each clusters marker genes were identified using the FindConservedMarkers function as implemented in the Seurat package (logfc.threshold > 0.25 and minPct > 0.25). Next, clusters were annotated with known cell types according to the Cell Marker database [37]. Differently expressed genes across samples were identified using the FindConservedMarkers function in Seurat (logfc.threshold > 0.25, minPct > 0.25, and Padj ≤ 0.05). Finally, pseudotime trajectory analysis was conducted with the R package Monocle2 [38].

### Isolation and purification of Lin^−^CD117.^+^ hematopoietic stem cells (HSCs)

C57BL/6 mice (aged 8 weeks) were euthanized by cervical and sterilized in 75% alcohol for 30 s, preferably allowing the fur of mice to be completely wetted. Next, the femur and tibia were removed and placed in a Petri dish containing pre-chilled PBS for cleaning. The attached muscle tissue was removed as cleanly as possible, and then the clean bone with muscle tissue removed was placed in pre-chilled PBS. Next, both ends of the tibia were cut and the bone marrow was flushed out with a 1-mL syringe until the bone turned white. Bone marrow cells were gently dispersed, and the resulting bone marrow monocyte PBS suspension was collected and filtered through a 40-µm cell sieve. Subsequently, the bone marrow was centrifuged at 440 × g for 5 min and the supernatant was discarded. Single bone marrow cells were resuspended in 5 mL of red blood cell lysate, and lysis was terminated with 5 mL of pre-cooled PBS after 1–2 min. Next, the cells were centrifuged at 440 × g for 5 min, the supernatant was discarded, and 5 mL of pre-cooled PBS was used to wash the cells. An aliquot was taken for counting. After negative selection of lineage cells using a mouse Lineage Cell Depletion Kit (Miltenyi Biotech), the cells were subjected to positive selection of CD117^+^ cells to obtain the target Lin^−^CD117^+^ HSCs.

### OP9/OP9-DL1 co-culture system

OP9/OP9-DL1 cells were co-cultured as previously described [20–22]. OP9 was co-cultured with Lin^−^CD117^+^ HSCs for directed differentiation into B cells, while OP9-DL1 was co-cultured with Lin^−^CD117^+^ HSCs for directed differentiation into T cells. Before preparing for differentiation, OP9/OP9-DL1 cells grown in log phase were seeded at a density of 3 × 10^4^ cells/well in 12-well plates, and 10^5^ Lin^−^CD117^+^ HSCs were added to each well after apposition. Lin^−^CD117^+^ HSCs were co-cultured with OP9/OP9-DL1 cells in 20% fetal bovine serum (FBS), 5% penicillin/streptomycin (P/S), 5 ng/mL recombinant mouse IL-7, and 5 ng/mL rmFit3-L in α-Minimum Essential Medium. After 12 d of differentiation into B cells, or 12 and 28 d of differentiation into T cells, control (Ctrl) and GA groups with a glycyrrhizic acid concentration of 3.125 µM were evaluated (*n* = 3 replicates per group).

### Cell preparation and flow cytometry analysis

Briefly, harvested mouse spleens were infiltrated with phosphate buffer, washed, lysed with lysis buffer, and filtered with a 70 µm cell strainer. Fresh blood samples were collected in heparin sodium tubes. Flow cytometry was used to directly quantify numbers of T cells (CD3 +) and B cells (CD45R/B220 +) in the spleen.

According to Roxanne Holmes' research [20], OP9-DL-1 cells are bone marrow-derived stromal cells that peripherally express the Notch ligand Delta-like 1 to support in vitro differentiation and development of T cells. T cell development proceeds through CD44 and CD25 yin-yang selection (DN phase changes) to yield CD44-CD25- (double-negative) cells, followed by CD4 + CD8 + double-positive cells, which ultimately mature into CD4 + /CD8 + single-positive cells. OP9 cells support in vitro differentiation and development of B cells [21]. At the end of co-culture, the upper cells were collected, rinsed 2–3 times with PBS, processed immunocytochemically, and quantified by flow cytometry (Fig. S9). Numbers of DN1 (CD44 + CD25-), DN2 (CD44 + CD25 +), DN3 (CD44-CD25-), DN4 (CD44-CD25-), T cells (CD4 + /CD8 +), B cells (CD45R/B220 +) and CD11b + cells were assessed. The optimal working concentration for all antibodies was 1 μg/mL. Data were analyzed with FlowJo software (Ashland, OR).

### Label-free quantitative (LFQ) proteomics

Following extraction of total protein from PBMCs, a bicinchoninic acid assay was used to quantify protein and divide it into six 100-µg aliquots. For each group of three aliquots, methanol was added to the control group and the small molecule GA was added to the treatment group. After incubating samples at 25 °C for 10 min, PK (1:100) was added for incubation at 25 °C for 5 min. Subsequently, samples were transferred to a water bath with a temperature greater than 95 °C to completely inactivate PK. Next, samples were cooled and placed at room temperature for 5 min. After adding 2% Sodium deoxycholate (DOC) and 200 mM Ammonium bicarbonate (ABC), DL-Dithiothreitol (DTT) was added to samples and incubated at 37 °C for 30 min. Next, Iodoacetamide (IAA) was added and incubated at room temperature (protected from light) for 45 min. Subsequently, trypsin was added according to the ratio (1:50) and digested overnight in a 37 °C water bath. The following day, 50% Trifluoroacetic acid (TFA) was added to terminate the enzymatic termination and precipitation, and the enzymatically cleaved peptides were eluted. After elution, peptides were digested and resuspended in 100 µL of 0.1% Formic acid (FA) to yield a final concentration greater than 2 µg/µL. Finally, mass spectrometry was performed.

### nanoLC-MS/MS analysis

Two microliters of total peptide were taken from each sample and separated with an EASY-nLC1200 nano-UPLC Liquid Phase System (Thermo Fisher Scientific). Data were collected using a mass spectrometer (Q-Exactive HFX; Thermo Fisher Scientific) equipped with a nano-electrospray ion source. Separation was performed with a reversed-phase column (100 μm ID × 15 cm, Reprosil-Pur 120 C18-AQ, 1.9 μm). The mobile phase adopted the acetonitrile–water-formic acid system, in which mobile phase A was 0.1% formic acid-98% aqueous solution (2% in acetonitrile) and phase B was 0.1% formic acid-80% acetonitrile (20% in water). After the column was equilibrated with a 100% A phase, the sample was directly loaded onto the column and passed through the column ladder degree separation with a flow rate of 300 nL/min and gradient length of 120 min. Mobile phase B ratios were applied as follows: 5% for 2 min, 5%–22% for 98 min, 22%–45% for 16 min, 45%–95% for 2 min, and 95% for 2 min. Mass spectrometry analysis used a data-dependent acquisition mode with a total analysis time of 120 min and positive ion detection mode.

### Molecular docking

The binding of GA and S100A8 protein was simulated with CB-Dock (http://clab.labshare.cn/cb-dock/php/blinddock.php) and AutoDock software. Homology modeling was performed in SWISS-MODEL to predict the S100A8 protein structure with and without GA secondary structures. The CB-Dock computing program for AutoDock Vina was used to select the structure with highest score, as predicted by a total of 50 simulation positions according to the binding affinity sorting. The highest affinity group from the predicted results was put into PyMOL for visualization. The hydrogen bond formed between GA and S100A8 protein is the proposed binding site.

### Surface plasmon resonance (SPR) analysis of GA with S100A8

The binding ability of GA to S100A8 protein was measured by SPR on a BIAcore3000 system (GE Healthcare, Chicago, IL). The S100A8 protein was immobilized on a CM5 chip by its amine group. A 10-µL aliquot of 0.1 mg/mL S100A8 was injected into the flow cell at a rate of 5 µL/min. Successful immobilization of S100A8 was verified by adding approximately 5000 RU to the sensor chip. No S100A8 was injected into the first flow cell as a control. GA was diluted in buffer containing 20 mM Tris–HCl, 150 mM NaCl, and 1 mM TCEP (pH 7.2). After fixation, varying dilutions of GA were injected at 30 µL/min for 3 min. After sample injection, the flow buffer was allowed to dissociate by passing over the sensor for 3 min. The sensor surface was regenerated by injecting 20 µL of 10 mM glycine–HCl solution (pH 2.25). Generated data were analyzed at 25 °C using Bioassessment Software 4.1.1 (GE Healthcare).

### RNA sequencing (RNA-seq)

Total RNA from samples was extracted to create an RNA-seq library, which was analyzed by BGI USA (Cambridge, MA) using a BGISEQ-500 sequencer. Raw sequencing reads were cleaned by removing reads containing aptamer or poly-N sequences, and reads with low-quality base ratios. Afterwards, clean reads were obtained and stored in FASTQ format. Clean reads were mapped to the reference genome using the HISAT2 (v2.0.4) [39]/Bowtie2 (v2.2.5) [40] tool. Gene expression levels were calculated using RESM software [41] and normalized to determine gene expression. Heat maps were plotted using Graphpad Prism 8 (GraphPad, San Diego, CA). To gain insight into phenotypic variation, Kyoto Encyclopedia of Genes and Genomes (KEGG, https://www.kegg.jp/) enrichment analysis of annotated differentially expressed genes was performed by Phyper (https://en.wikipedia.org/wiki/Hypergeometric_distribution) based on hypergeometric tests. Terms and pathways were corrected for significance levels by Bonferroni's strict threshold (Q value ≤ 0.05).

### qRT-PCR

Total RNAs were extracted from cells using TRIzol (Invitrogen, Carlsbad, CA) according to the manufacturer’s instructions. cDNA was prepared using reverse transcriptase (Takara, Kusatsu, Japan) according to the manufacturer's protocol. qRT-PCR reactions were performed using SYBR green-fluorescent dye and the primer sequences below.

### sgRNA and CRISPR/Cas9 vector construction, virus generation, and transduction

sgRNA of S100A8 (sgRNA1F, 5'-caccgGACATCAATGAGGTTGCTCA-3'; sgRNA1R, 5'-aaacTGAGCAACCTCATTGATGTCc-3'; sgRNA2F, 5'-caccgGTCCTCAGTTTGTGCAGGTG-3'; and sgRNA2R, 5'-aaacCACCTGCACAAACTGAGGACc-3'; http://www.e-crisp.org/E-CRISPR) were cloned into the Lenti-CRISPR v2 vector following linearization by *BsmB1* enzyme. Lentiviruses were generated by transiently transfecting 293 T cells with lentiviral plasmids VSVG and REV, and Pmdl packaging plasmids using Lentifit™ (HanBio Technology, Shanghai, China). Lentiviral particles were collected using ultracentrifugation. Lin^−^CD117^+^ HSCs were cultured in Ham's F-12 Nutrient Mixture with 1 × Penicillin–streptomycin–glutamine (PSG), 10 mM (4-(2-hydroxyethyl)-1-piperazineethanesulphonic acid) (HEPES), 1 mg/mL Polyvinyl alcohol (PVA), 1 × Insulin–transferrin–selenium–ethanolamine (ITSX), 100 ng/mL thrombopoietin (TPO), and 10 ng/mL stem cell factor for 24 h before adding lentiviral particles. Particles were incubated at room temperature for 15–30 min and then added to cells in culture dishes for incubation at 37 °C overnight. Mouse embryonic fibroblasts (MEF) cultured in Dulbecco’s Modified Eagle Medium with 10% FBS and 5% P/S were infected with virus by the 1/2 small-volume infection method. After 4 h of infection, the medium was replenished with complete culture volume. After 24 h of infection, the culture medium was replaced with fresh complete medium for continued culture. Transfected cells were positively screened with 2 μg/mL of puromycin.

### Immune Reconstruction (IR)

B-NDG mice are severely immunodeficient with severe deficiencies of T, B, and NK cells, which could be reconstituted with Lin^−^CD117^+^ HSCs modified with lentiviral particles in vitro. Lin^−^CD117^+^ HSCs were infected with LV201 and OEa8 lentiviral particles, and 10^5^ particles/mouse were intravenously injected into the tail of B-NDG mice. One week after IR, mice in good health were administered GA (5 mg/kg every 2 d, lasts 30 days) by tail vein injection. Experiments were divided into five groups: non-IR, IR + PBS, IR + GA, IR-OEa8 + PBS, and IR-OEa8 (PM69) + GA.

### Open field test (OFT)

A square box (50 × 50 × 30 cm) was used to evaluate the anxiety of mice. Anxious mice prefer to stay in the corner of the box and make stereotyped movements along the sides. At the beginning of the experiment, mice were placed in the same corner of the box, and the trajectory of mice in the box was tracked and recorded for 15 min. The anxiety of mice was judged by observing the time mice spent in the central area.

### New object recognition (NOR)

Novel object recognition experiments use the rodent's natural instinct to approach and explore novel objects to test the behavioral sensitivity of the animal's recognition memory. The NOR experiment was divided into three phases: the adaptation period, familiarization period, and constant-temperature test period. Adaptation period: The new object was a square black box, and mice were put into one corner of the black box and allowed to explore the box freely for 10 min to adapt and reduce the stress of mice to the unfamiliar environment. Familiarization period: Two identical objects (A1 and A2) were placed diagonally across the bottom of the black box at a certain distance from the side, and mice were put into one corner of the black box and allowed to freely explore and familiarize with these two objects for 10 min. After familiarization, mice were put back into the cage for familiarization and tested after a certain period of time. Test period: A1 of the two identical objects was replaced in the black box with new object B. Mice were placed into the same corner of the black box they were put into during adaptation and familiarization, and allowed to explore the new object freely for 5 min. Video recordings were used to track the exploration times of mice for A2 and B. Preference for the new object was quantified by "discrimination index", expressed as D2 and calculated according to the formula: D2 = N/(F_N_ + F), where "N" is the exploration time of mice for B, "F_N_" is the exploration time of mice for B, and "F" is the exploration time of mice for A2.

### Statistical analysis

Data were statistically analyzed using Graphpad Prism 8.0 and expressed as mean ± standard error of the mean (SEM). Statistical comparisons between two groups were performed using an unpaired t-test. Probability values less than 0.05 were considered statistically significant.

## Supplementary Information

**Additional file 1: Figure S1.** Single-cell clustering analysis of PBMCs from mice to evaluate changes in immune cell proportions during aging. (A) Clustering of single cells in 8-week-old (young) mice. (B) Clustering of single cells in 16-month-old (aged) mice. (C) Comparison of clustering of single cells in young and aged mice. (D) Relative population abundances of cell types in young and aged mice. (E) Kyoto Encyclopedia of Genes and Genomes (KEGG) analysis of differentially expressed genes in Cluster 1 (T cells, *Igfbp4* as marker gene). (F) KEGG analysis of differentially expressed genes in Cluster 2 (T cells, *Cd8b1* as marker gene). (G) KEGG analysis of differentially expressed genes in Cluster 7 (basal cells, *Dapl1* as marker gene).

**Additional file 2: Figure S2.** Proposed temporal developmental trajectory of PBMCs from single-cell sequencing of GA-treated aged mice. (A) Temporal developmental trajectory constructed from PBMCs of GA-treated aged mice. a indicates the temporal developmental trajectory, whereby the darker color represents the earlier developmental stage after temporal differentiation; b indicates the position of the developmental stage on the developmental trajectory; and **c** indicates the distribution of each cell taxon on the developmental trajectory after clustering of single-cell sequencing results. (B) Clustering of single cells in aged + GA mice. (C) Cell clusters were divided into three stages according to the proposed temporal developmental stage. (D) Expression of *S100A8* in each cell cluster.

**Additional file 3: Figure S3.** GA increased the proliferation of T- and B-cell subsets in vitro. (A) Representative FACS plots of CD3^+^ T cells and CD45R/B220^+^ B cells in spleen cells of GA-treated C57BL/6 mice in vitro. (B) Bar graph of T- and B-cell statistics of spleen cells from GA-treated C57BL/6 mice in vitro. Data represent mean ± SEM. *n* = 9, ****P < 0.0001.

**Additional file 4: Figure S4**. RNA-seq of GA-treated Lin^−^CD117^+^ HSCs in vitro. (A) Differentially expressed genes in GA versus control; *S100A8* and *S100A9* were upregulated. (B) KEGG pathway enrichment map of GA and control differentially expressed genes. (C) Bar graph showing upregulated S100A8 expression in GA-treated Lin^−^CD117^+^ HSCs in vitro by qRT-PCR. (D) Bar graph showing upregulated S100A9 expression in GA-treated Lin^−^CD117^+^ HSCs in vitro by qRT-PCR. Data represent mean ± SEM. *n* = 3, *P < 0.05.

**Additional file 5: Figure S5.** GA reduced *S100A8* expression levels in PBMCs. (A) Cell clustering maps of single-cell sequencing for PBMCs of young, aged, and aged-GA mice. (B) Annotated cell clustering maps of single-cell sequencing for PBMCs in young, aged, and aged-GA mice. (C) Expression levels of *S100A8* in young, aged, and aged-GA mice.

**Additional file 6: Figure S6**. RNA-seq of MEFs with point mutation (E69 and N67) and knockdown of *S100A8*. (A) Venn diagram of differentially expressed genes for *S100A8* point mutations E69 and N67 compared with control. (B) Bar graph showing qRT-PCR validation of *S100A8* knockdown in MEFs. (C) Differentially expressed genes in GA-treated *S100A8*-knockdown MEFs. (D) KEGG pathway enrichment map of differentially expressed genes in GA-treated *S100A8*-knockdown MEFs. (E) Enrichment map of IL-17 signaling pathway gene expression profiles with barcodes indicating the location of genes in each gene set. NES, normalized enrichment score. Data represent mean ± SEM. *n* = 3, *P < 0.05.

**Additional file 7: Figure S7.** Representative FACS plots and bar graphs showing CD45R/B220^+^ B cells in the spleens of B-NDG mice in non-IR + PBS, IR + PBS, IR + GA, IR-OEa8 + PBS, and IR-OEa8 (PM69) + GA groups.

**Additional file 8: Figure S8.** GA is competitively bound to S100A9 in binding S100A8. (A) Binding affinity of SPR COOH and S100A8-S100A9 interactions. S100A8 was immobilized on a COOH chip with S100A9 concentrations (from top to bottom) of 800, 400, 200, 100, and 50 μM. (B) GA affects the binding affinity of S100A9 in 400 nM S100A8. Concentrations of GA (from top to bottom) were 1 mM, 500 μM, 250 μM, and 125 μM; additionally, 400 nM S100A8 was evaluated.

**Additional file 9: Supplementary Fig. 9**. T cell differentiation development flow cytometry gating strategy map. APC-CD25, PE-CD44, PE-CY7 Tm-CD11b. DN1 (CD44 + CD25-), DN2 (CD44 + CD25 +), DN3 (CD44-CD25-), DN4 (CD44-CD25-), and CD11b + cell.

## Data Availability

The data that support the findings of this study are available from the corresponding author upon reasonable request. RNA sequencing data are deposited in Gene Expression Omnibus (GEO) with accession number GSE216600.

## Abbreviations

GA: Glycyrrhizic acid
S100A8: S100 calcium-binding protein 8
SASP: Senescence-associated secretory phenotype
MS: Mass spectrometry
KEGG: Kyoto Encyclopedia of Genes and Genomes
NK: Natural killer
IL: Interleukin
TNF-α: Tumor necrosis factor α
NF-κB: Nuclear factor κB
PBMCs: Peripheral blood mononuclear cells
HSCs: Hematopoietic stem cells
GSEA: Gene Set Enrichment Analysis
AML: Acute myeloid leukemia
PBS: Phosphate-buffed saline
qPCR: Quantitative PCR
UMAP: Uniform Manifold Approximation and Projection
FBS: Fetal bovine serum
DTT: Dithiothreitol
IAA: Iodoacetamide
TFA: Trifluoroacetic acid
RNA-seq: RNA sequencing
HEPES: 4-(2-Hydroxyethyl)-1-piperazineethanesulphonic acid
ITSX: Insulin–transferrin–selenium–ethanolamine
TPO: Thrombopoietin
MEF: Mouse embryonic fibroblasts
IR: Immune Reconstruction
OFT: Open field test
NOR: New object recognition
SEM: Standard error of the mean
