# Supplementary figures and images for: Glycyrrhizic acid inhibits myeloid differentiation of hematopoietic stem cells by binding S100 calcium binding protein A8 to improve cognition in aged mice
Source: Immun Ageing. 2023 Mar 11;20:12. doi: 10.1186/s12979-023-00337-9 (PMC10007777; doi:10.1186/s12979-023-00337-9)

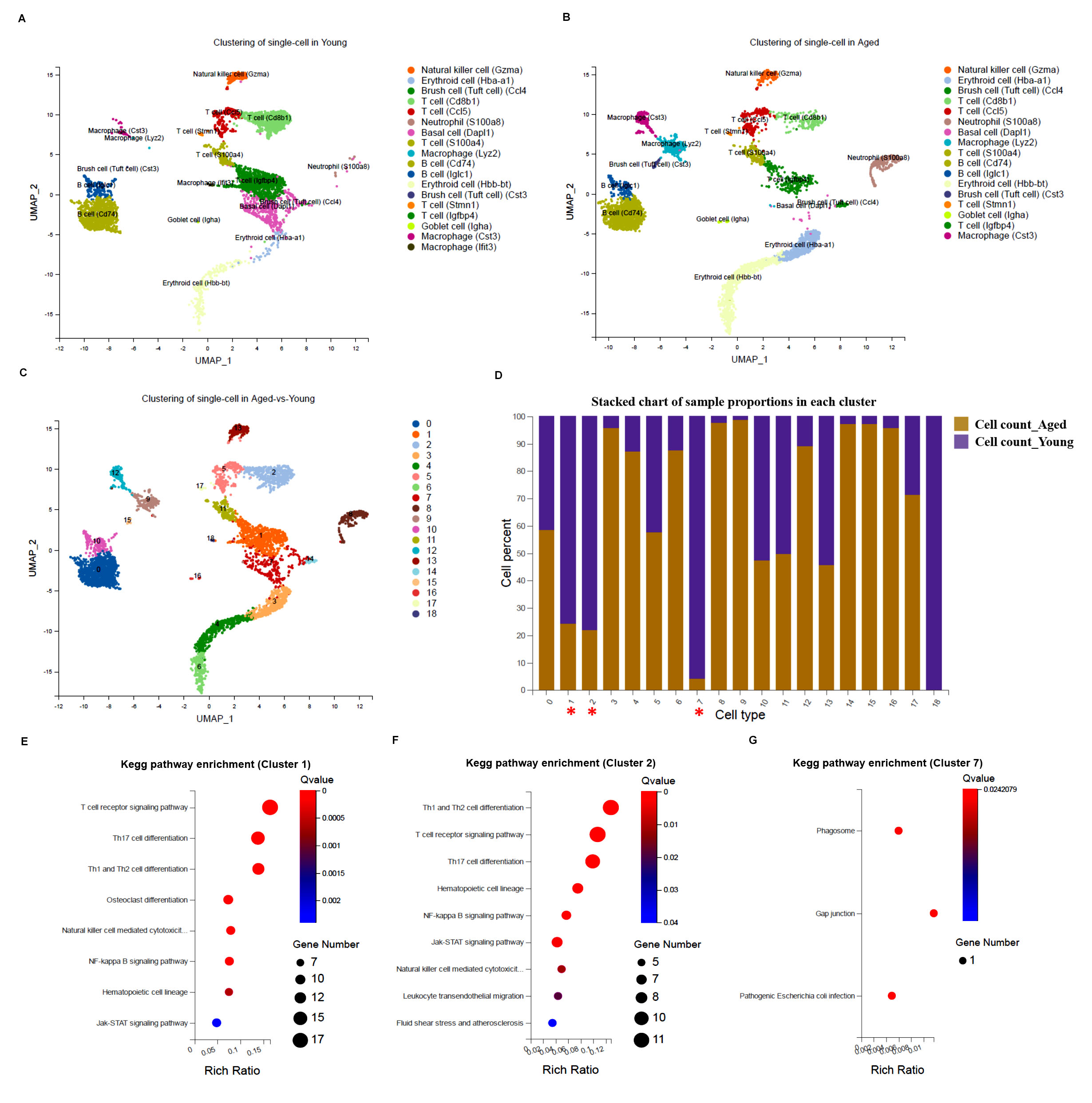

Supplement: Supplementary file 1 — Additional file 1: Figure S1. Single-cell clustering analysis of PBMCs from mice to evaluate changes in immune cell proportions during aging. (A) Clustering of single cells in 8-week-old (young) mice. (B) Clustering of single cells in 16-month-old (aged) mice. (C) Comparison of clustering of single cells in young and aged mice. (D) Relative population abundances of cell types in young and aged mice. (E) Kyoto Encyclopedia of Genes and Genomes (KEGG) analysis of differentially expressed genes in Cluster 1 (T cells, Igfbp4 as marker gene). (F) KEGG analysis of differentially expressed genes in Cluster 2 (T cells, Cd8b1 as marker gene). (G) KEGG analysis of differentially expressed genes in Cluster 7 (basal cells, Dapl1 as marker gene). [file 12979_2023_337_MOESM1_ESM.jpg]

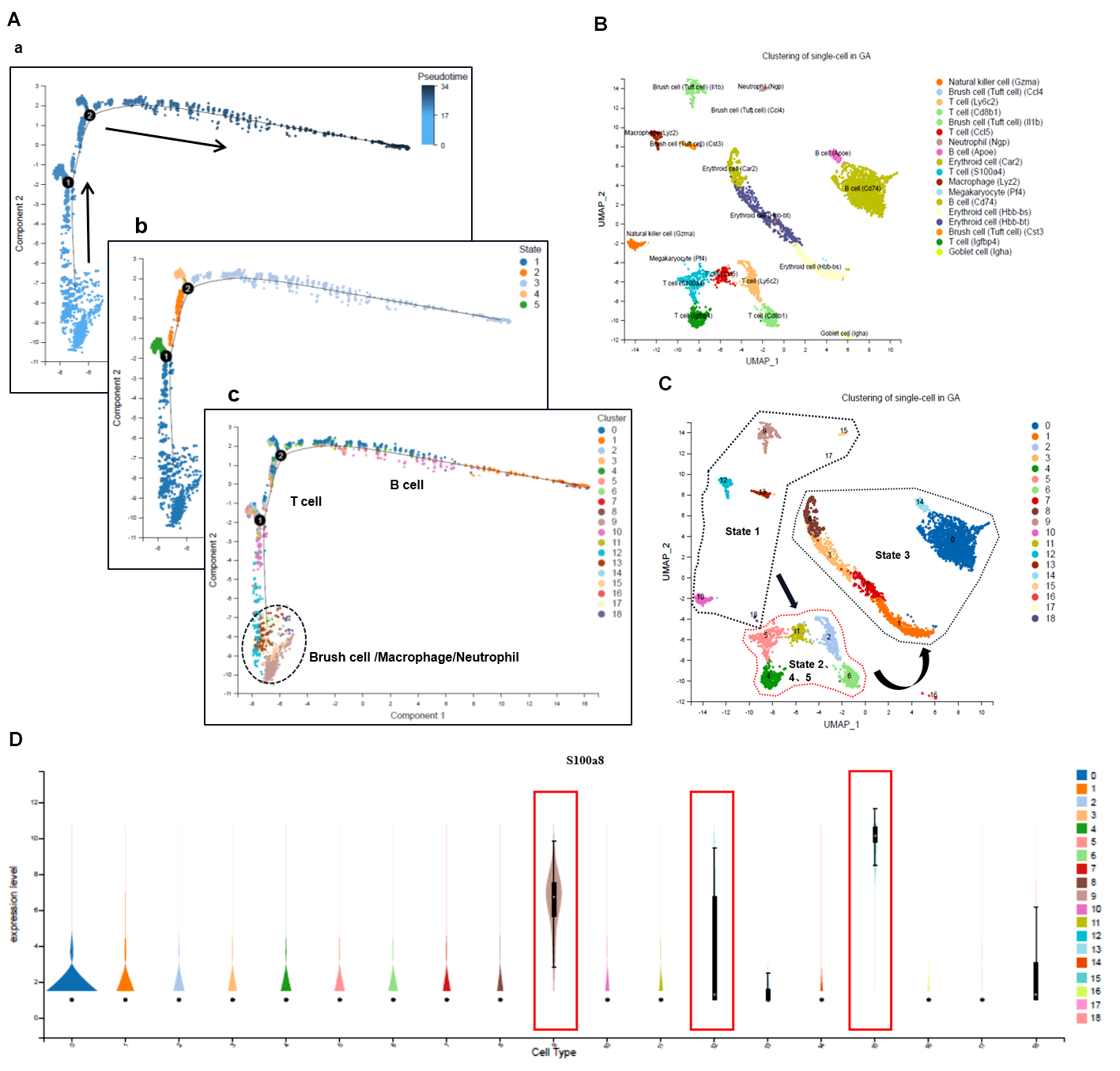

Supplement: Supplementary file 2 — Additional file 2: Figure S2. Proposed temporal developmental trajectory of PBMCs from single-cell sequencing of GA-treated aged mice. (A) Temporal developmental trajectory constructed from PBMCs of GA-treated aged mice. a indicates the temporal developmental trajectory, whereby the darker color represents the earlier developmental stage after temporal differentiation; b indicates the position of the developmental stage on the developmental trajectory; and c indicates the distribution of each cell taxon on the developmental trajectory after clustering of single-cell sequencing results. (B) Clustering of single cells in aged + GA mice. (C) Cell clusters were divided into three stages according to the proposed temporal developmental stage. (D) Expression of S100A8 in each cell cluster. [file 12979_2023_337_MOESM2_ESM.tif]

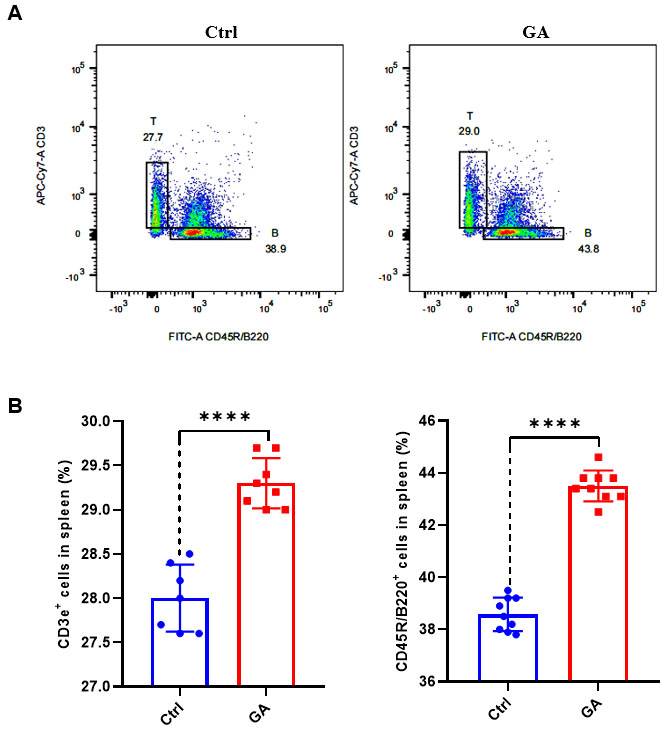

Supplement: Supplementary file 3 — Additional file 3: Figure S3. GA increased the proliferation of T- and B-cell subsets in vitro. (A) Representative FACS plots of CD3+ T cells and CD45R/B220+ B cells in spleen cells of GA-treated C57BL/6 mice in vitro. (B) Bar graph of T- and B-cell statistics of spleen cells from GA-treated C57BL/6 mice in vitro. Data represent mean ± SEM. n = 9, ****P < 0.0001. [file 12979_2023_337_MOESM3_ESM.jpg]

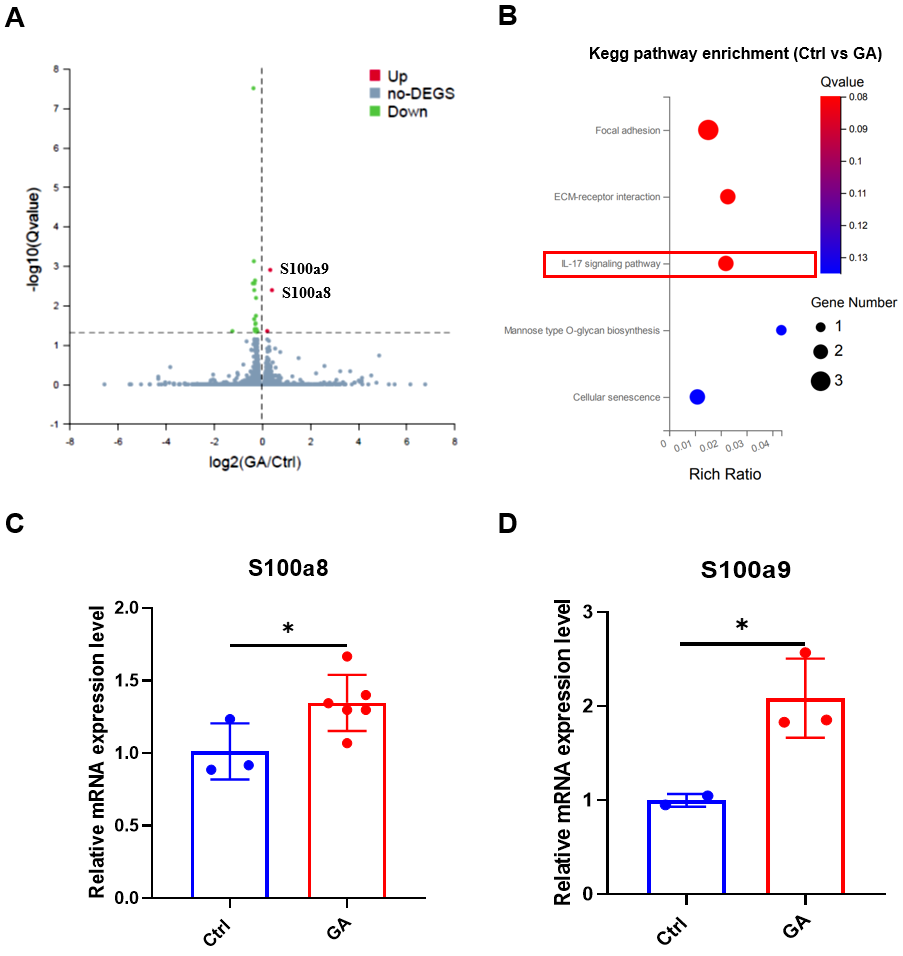

Supplement: Supplementary file 4 — Additional file 4: Figure S4. RNA-seq of GA-treated Lin−CD117+ HSCs in vitro. (A) Differentially expressed genes in GA versus control; S100A8 and S100A9 were upregulated. (B) KEGG pathway enrichment map of GA and control differentially expressed genes. (C) Bar graph showing upregulated S100A8 expression in GA-treated Lin−CD117+ HSCs in vitro by qRT-PCR. (D) Bar graph showing upregulated S100A9 expression in GA-treated Lin−CD117+ HSCs in vitro by qRT-PCR. Data represent mean ± SEM. n = 3, *P < 0.05. [file 12979_2023_337_MOESM4_ESM.tif]

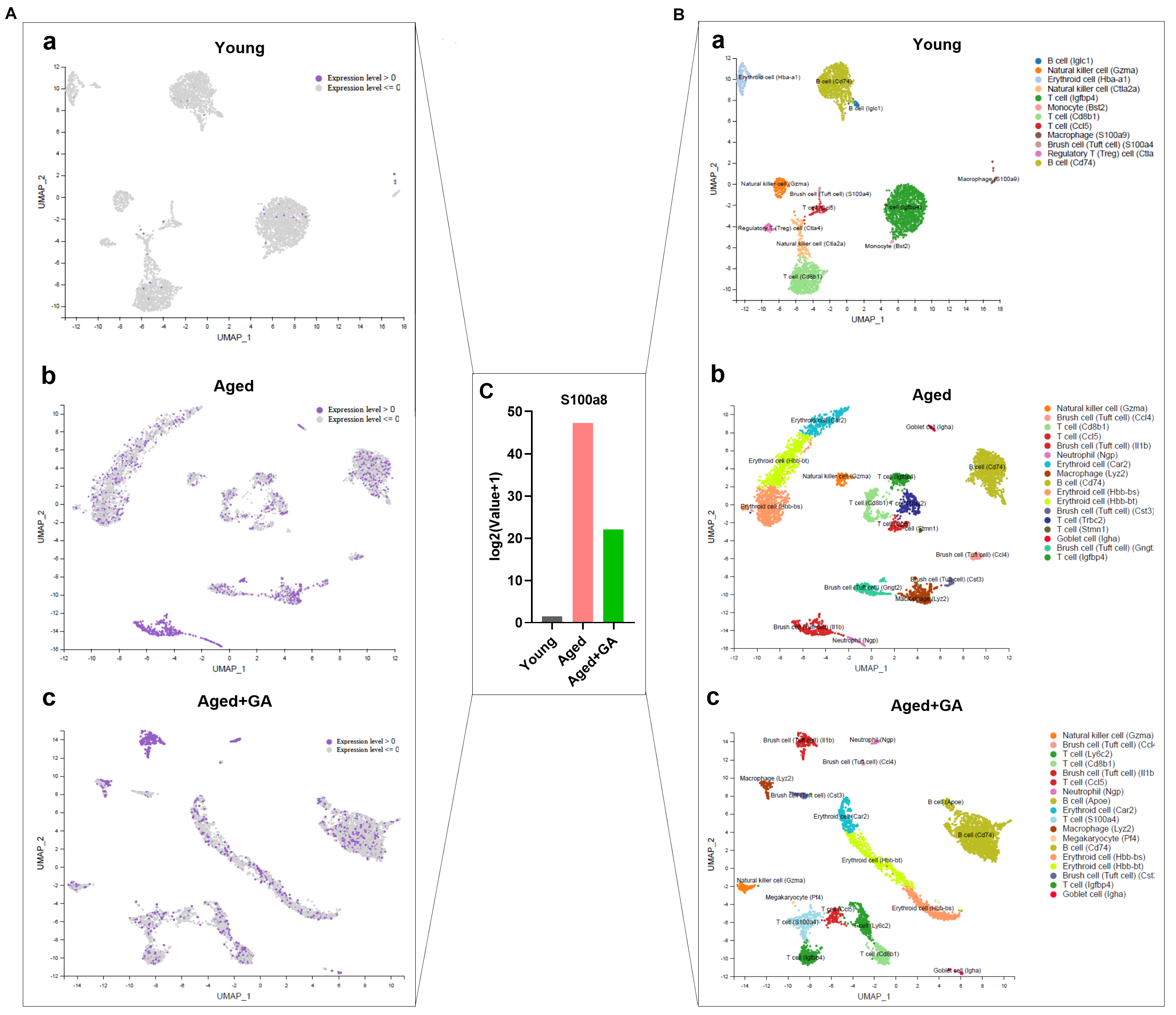

Supplement: Supplementary file 5 — Additional file 5: Figure S5. GA reduced S100A8 expression levels in PBMCs. (A) Cell clustering maps of single-cell sequencing for PBMCs of young, aged, and aged-GA mice. (B) Annotated cell clustering maps of single-cell sequencing for PBMCs in young, aged, and aged-GA mice. (C) Expression levels of S100A8 in young, aged, and aged-GA mice. [file 12979_2023_337_MOESM5_ESM.tif]

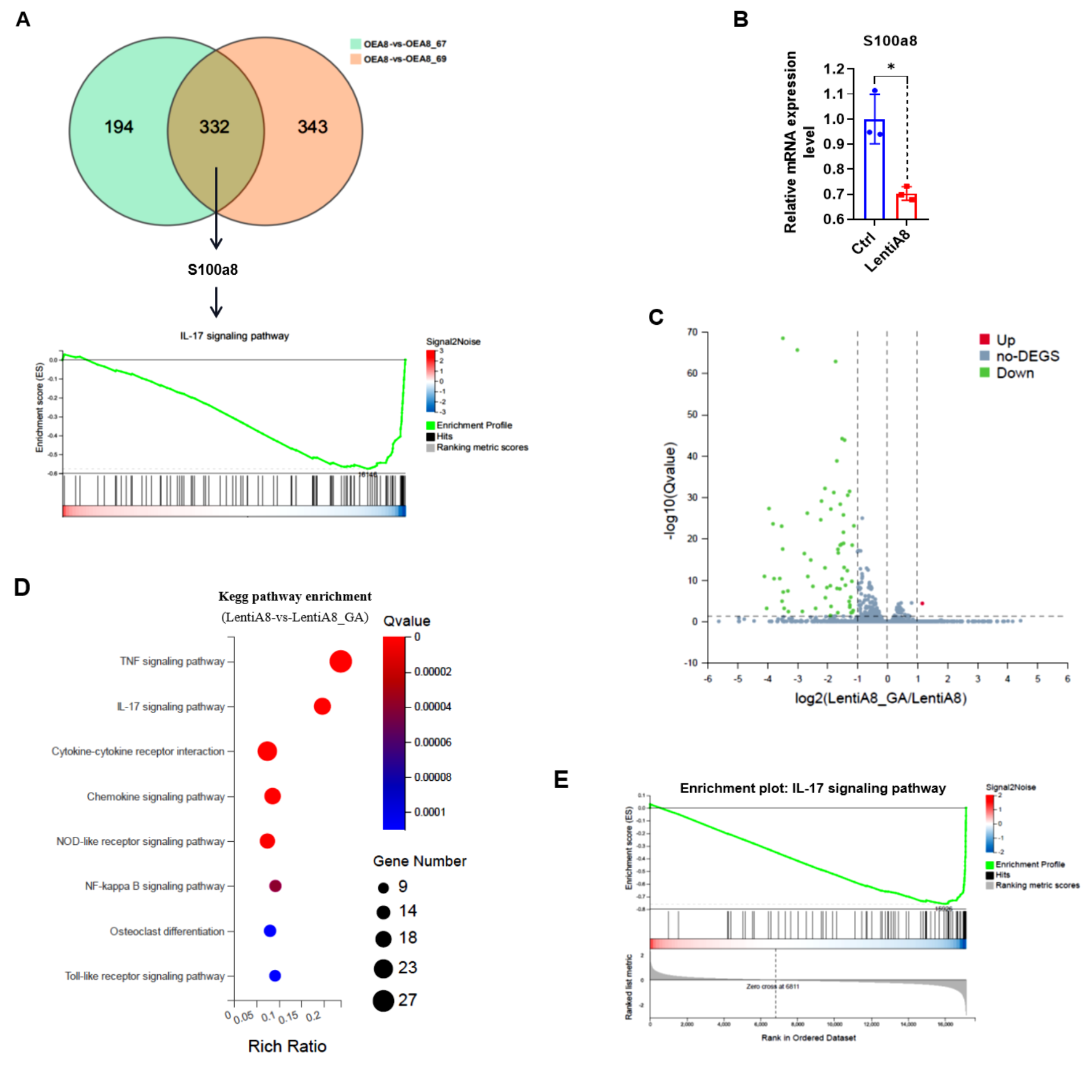

Supplement: Supplementary file 6 — Additional file 6: Figure S6. RNA-seq of MEFs with point mutation (E69 and N67) and knockdown of S100A8. (A) Venn diagram of differentially expressed genes for S100A8 point mutations E69 and N67 compared with control. (B) Bar graph showing qRT-PCR validation of S100A8 knockdown in MEFs. (C) Differentially expressed genes in GA-treated S100A8-knockdown MEFs. (D) KEGG pathway enrichment map of differentially expressed genes in GA-treated S100A8-knockdown MEFs. (E) Enrichment map of IL-17 signaling pathway gene expression profiles with barcodes indicating the location of genes in each gene set. NES, normalized enrichment score. Data represent mean ± SEM. n = 3, *P < 0.05. [file 12979_2023_337_MOESM6_ESM.tif]

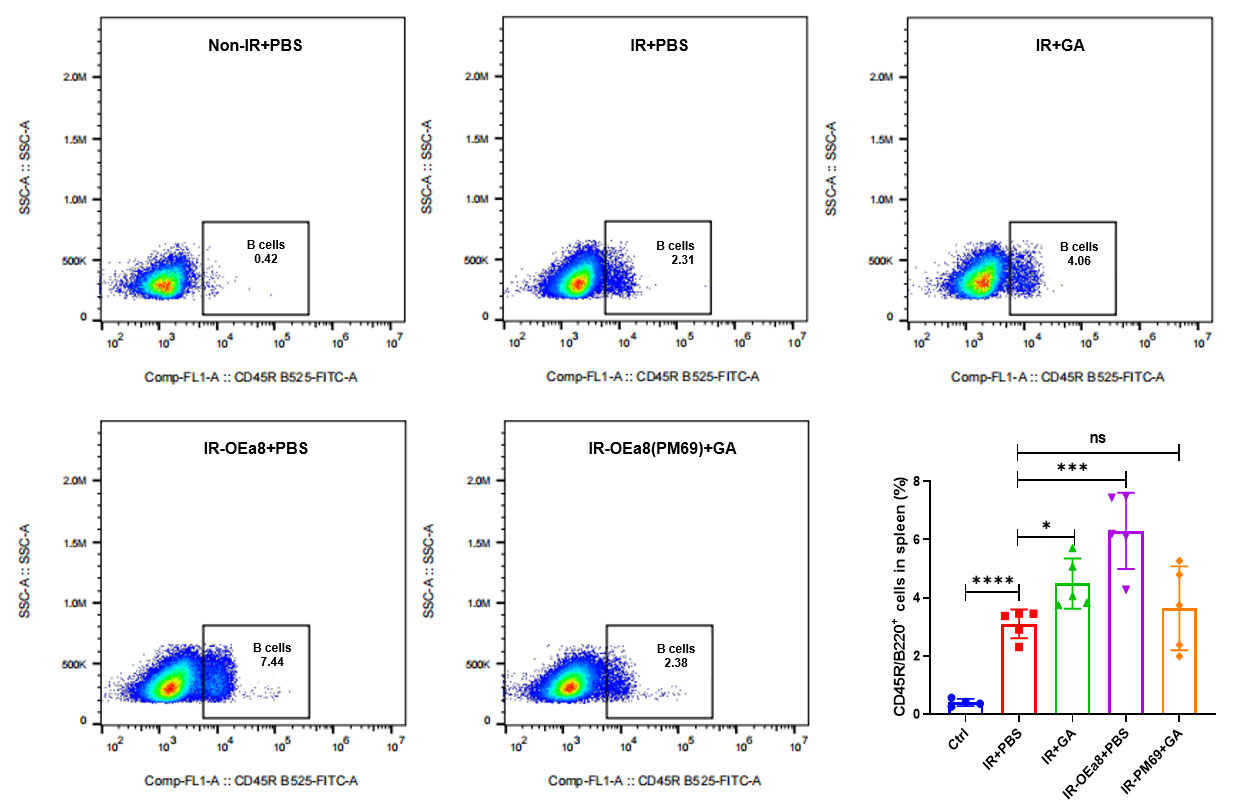

Supplement: Supplementary file 7 — Additional file 7: Figure S7. Representative FACS plots and bar graphs showing CD45R/B220+ B cells in the spleens of B-NDG mice in non-IR + PBS, IR + PBS, IR + GA, IR-OEa8 + PBS, and IR-OEa8 (PM69) + GA groups. [file 12979_2023_337_MOESM7_ESM.jpg]

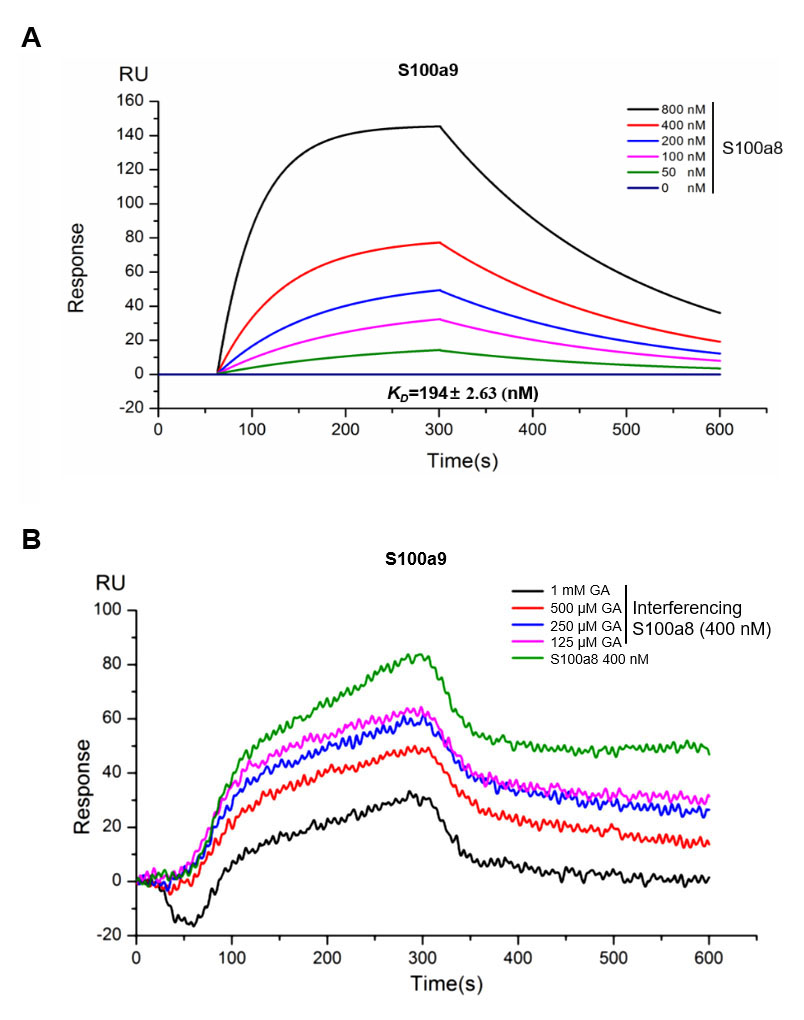

Supplement: Supplementary file 8 — Additional file 8: Figure S8. GA is competitively bound to S100A9 in binding S100A8. (A) Binding affinity of SPR COOH and S100A8-S100A9 interactions. S100A8 was immobilized on a COOH chip with S100A9 concentrations (from top to bottom) of 800, 400, 200, 100, and 50 μM. (B) GA affects the binding affinity of S100A9 in 400 nM S100A8. Concentrations of GA (from top to bottom) were 1 mM, 500 μM, 250 μM, and 125 μM; additionally, 400 nM S100A8 was evaluated. [file 12979_2023_337_MOESM8_ESM.jpg]

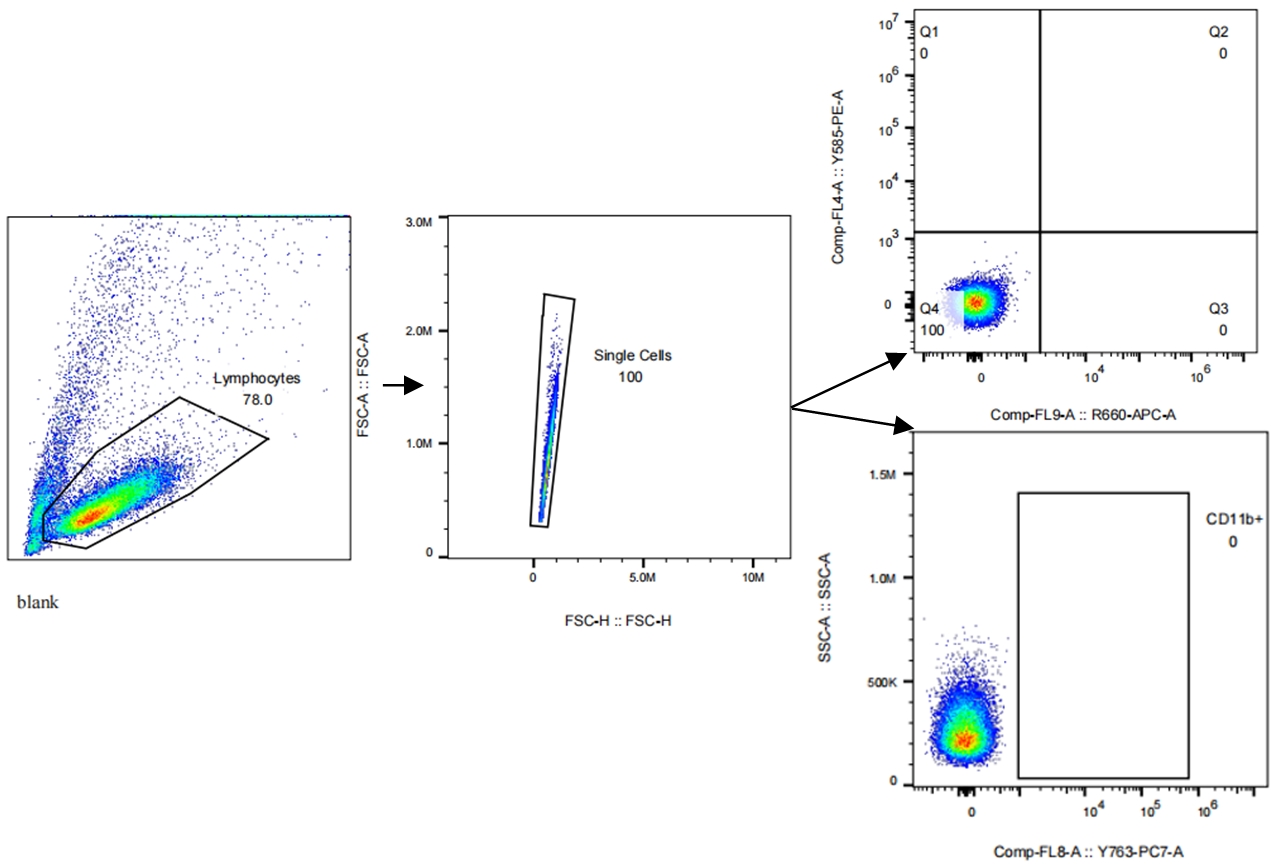

Supplement: Supplementary file 9 — Additional file 9: Supplementary Fig. 9. T cell differentiation development flow cytometry gating strategy map. APC-CD25, PE-CD44, PE-CY7 Tm-CD11b. DN1 (CD44 + CD25-), DN2 (CD44 + CD25 +), DN3 (CD44-CD25-), DN4 (CD44-CD25-), and CD11b + cell. [file 12979_2023_337_MOESM9_ESM.tif]
